# Large language model enhanced public opinion monitoring for China’s portable energy market: A data driven analysis of social media comments

**DOI:** 10.1371/journal.pone.0357018

**Published:** 2026-09-16

**Authors:** Yun Zhang, Weikang Xie, Xiao Li, Wei Liao, Tao Shu, Jixian Zhou, Jun Wang

**Affiliations:** 1 College of Artificial Intelligence, Chengdu University of Information Technology, Chengdu, China; 2 School of Software Engineering, Chengdu University of Information Technology, Chengdu, China; 3 School of Computing and Artificial Intelligence, Southwestern University of Finance and Economics, Chengdu, China; 4 School of Management Science and Engineering, Southwestern University of Finance and Economics, Chengdu, China; Guilin University of Technology, CHINA

## Abstract

Portable power banks, as a cornerstone of China’s mobile and shared energy market, are closely integrated into daily life and work. The recent, ongoing exposure of safety incidents has led to stricter control measures in specific industries and regions, and even to the revision of relevant administrative management standards, posing a significant challenge to an industry with over a decade of rapid growth. The expectations of the public and government administrative management for product safety, reliability, and service quality have risen rapidly. Therefore, real-time public opinion monitoring is vital for supporting technological innovation, safety governance, and the sustainable development of the industry. However, traditional opinion mining methods struggle to interpret short, noisy, and user-generated data and often lack generalizable semantic understanding. This study therefore develops an LLM-driven framework that first clusters LLM-augmented multi-view representations via graph-based consensus spectral clustering to discover latent topics, followed by multi-label topic assignment and aspect-based sentiment analysis through multi-LLM consensus voting. A total of 47,023 public comments were collected from China’s leading social media platforms, yielding 12 key discussion topics covering electrical safety, charging performance, device durability, shared rental experiences, and emerging magnetic attachment design. The results show that there are strong negative views on electrical safety (86.1%) and the convenience of shared rental services (78.4%), while the views on magnetic connection innovation are mainly positive (76.6%). The topic-event correlation analysis further indicates that opinion fluctuations are closely related to pricing disputes and security-related events. This study demonstrates the effectiveness of LLM-enhanced semantic modeling in public opinion monitoring in the consumer electronics energy field. The results offer reference value for emerging industry market norms under discussion, while the proposed framework can further support the sustainability of portable energy market development by guiding product optimization, risk early warning, and data-informed regulatory strategies.

## 1 Introduction

With the accelerating digitalization of daily life, mobile electronic devices have become indispensable in modern society. As the use of smartphones, tablets, wearable devices, and outdoor digital products grows, the demand for portable and reliable energy supply systems has increased. Portable power banks, as the core component of mobile energy solutions, play a crucial role in ensuring continuous power availability and maintaining device operability in diverse contexts—ranging from individual daily use to shared rental, outdoor recreation, and emergency response scenarios. During the past decade, these devices have evolved from simple battery extensions to intelligent consumer energy products that integrate safety controls, high-efficiency charging, and intelligent management features.

Globally, the portable power bank industry continues to expand. In 2024, its market value was estimated at USD 18.59 billion, with the Asia–Pacific region leading growth and innovation [1]. China, in particular, ranks first worldwide in both production and consumption, with an estimated market size of USD 1.046 billion, driven by consumer upgrading, techno-logical innovation, and diversified product designs. As the dominant manufacturer and exporter, China’s portable power bank sector reflects the nation’s broader progress in energy storage technology, material innovation, and the integration of smart electronics [2].

As a typical portable energy supply device, the development of power banks is closely linked to advances in battery technology. With continuous progress in energy storage materials and electronic control technologies, the energy supply systems of portable devices are evolving from conventional lithium-battery architectures toward high-power fast charging, intelligent management, safety compliance, and multi-form-factor designs [3–5]. Current mainstream products, in addition to employing lithium-ion cells, are also exploring emerging systems such as solid-state and sodium-ion batteries to enhance energy density and safety [3,6,7]; at the application level, the unification of interface standards, the adoption of magnetic attachment connections, and the proliferation of wireless charging technologies have further driven the intelligent advancement and scenario expansion of power bank products [4].

The rapid market expansion, however, has generated a diverse and sometimes polarized public discourse. Positive opinions emphasize performance, design aesthetics, and technological sophistication, while negative sentiment often centers on product quality, safety risks, and service reliability. For instance, issues such as battery overheating, shared-rental malfunction, and after-sales complaints frequently dominate online discussions [8,9]. Understanding these opinions is essential for optimizing product design, improving safety governance, and fostering sustainable industrial growth.

Meanwhile, external industry data (see Table 1) indicates that regulatory requirements and safety risks surrounding mobile power supplies have increased together in recent years. On the one hand, relevant regulatory bodies have continuously strengthened entry and compliance requirements for batteries and portable power supply products. On the other hand, there have been multiple product recalls, usage restrictions, and management issues involving mobile power supplies on public transportation, attracting widespread public attention at different stages. The tightening of policy reviews and accumulation of risk events have exacerbated public concerns about product reliability and potential hazards. These issues continue to be discussed in public spaces [10].

**Table 1:** Timeline of policy announcements and market events in the portable power bank sector.

Date	Event category	Policy / Event description
21 Jul 2023	National policy announcement	The State Administration for Market Regulation issued an official notice announcing the implementation of CCC certification management for lithium-ion batteries, battery packs, and portable power banks. The China Compulsory Certification (CCC) system is a mandatory market-access regime enforced by the Chinese government.
1 Aug 2023	Policy implementation	Official implementation of CCC certification requirements for lithium-ion batteries, battery packs, and portable power banks.
1 Aug 2024	Policy deadline	Lithium-ion batteries, battery packs, and portable power banks without valid CCC certificates and certification marks are prohibited from being manufactured, sold, imported, or used in any commercial activities.
Mid Jun 2025	Market safety crisis	Due to incidents of power bank spontaneous combustion, several universities in Beijing banned the use of ROMOSS power banks, drawing significant public attention.
16 Jun 2025	Brand recall	ROMOSS announced the recall of more than 491,000 portable power bank units with safety risks.
20 Jun 2025	Brand recall	Anker Innovations initiated a proactive recall of over 712,000 units of its basic-series portable power banks due to potential safety hazards in certain batches.
21 Jun 2025	Regulatory action	Media reports indicated that the CCC certificates of several portable power bank brands had been suspended.
26 Jun 2025	Civil aviation regulation release	The Civil Aviation Administration of China issued an emergency notice announcing that, starting June 28, portable power banks without CCC marks, with unclear labeling, or belonging to recalled models/batches would be prohibited on domestic flights.
27 Jun 2025	Public attention peak	Topics related to the new power bank regulations topped trending lists across major social media platforms, marking a peak in public attention.
28 Jun 2025	Civil aviation regulation enforcement	Multiple airports began enforcing the new power bank regulations, resulting in the confiscation of large numbers of passengers’ non-compliant devices.
1 Jul 2025	Policy clarification	Railway and metro authorities clarified that inspection procedures had not changed significantly and that power banks without CCC markings were not banned from entry.
2 Jul 2025	Regulatory response	The Civil Aviation Administration responded to concerns about confiscated power banks, stating that self-disposal, temporary storage, mailing services, and appropriate disposal for unclaimed items would be arranged.
6 Jul 2025	Brand impact	ROMOSS announced a six-month suspension of production and operations.
Jul 2025	National standard drafting initiated	The National Technical Committee for Electronic Product Safety Standardization (TC588), under the guidance of the Ministry of Industry and Information Technology, established a drafting group for the mandatory national standard *Safety Technical Specification for Portable Power Banks*.

*Note:* Data sourced from Civiw [10].

Prior studies, which are often grounded in frameworks such as the Value Adoption Model (VAM), suggest that perceived usefulness and ease of use promote adoption, whereas perceived risk and cost reduce user trust [11]. Consumers generally evaluate power banks based on criteria such as capacity, charging efficiency, and brand reliability [8], while psychological factors, such as “low-battery anxiety,” strongly influence behavior [12]. Furthermore, the unification of charging standards and safety certification across regions is shaping users’ expectations for product compatibility and compliance [13].

In general, existing research has revealed that the public has multidimensional concerns about functionality, trust, and safety [8,11]. Although these studies provide valuable insights into technological advancement and consumer psychology surrounding portable power banks, they do have certain limitations. As the forms and application scenarios of portable power banks continue to diversify, public discussions have expanded beyond specific brands or individual usage contexts [14–16], exhibiting a complex expressive structure that combines rational evaluation with emotional attitude [17–19]. Firstly, existing research has predominantly focused on performance parameters, quality metrics, or willingness-to-use analysis. Data have primarily been derived from questionnaires or interviews [8,11–13], which makes it challenging to capture diverse public expression in natural contexts. Secondly, research perspectives remain relatively fragmented. They often focus on shared power banks or specific consumption scenarios [9,11,20], and lack a systematic examination of diverse issues from a comprehensive perspective. Finally, in terms of technical methods, traditional text mining and topic modeling still rely on shallow word-frequency and clustering techniques. These methods make it hard to uncover latent semantic associations and emotional tendencies in short texts.

In recent years, user feedback and experiential data have become increasingly accessible through online platforms and social media, offering timely and wide-ranging information that complements traditional survey methods [21–23]. Such data provide a solid foundation for systematic analyses of public perception. Meanwhile, advances in machine learning and natural language processing (NLP), especially the large language models (LLMs), enable a more effective understanding of large-scale text data, supporting topic identification and sentiment recognition in public discourse [24–26].

Accordingly, this study proposes an LLMs-driven semantic analysis framework to investigate and monitor Chinese public opinion on portable power banks using social media comments. Specifically, the study (1) constructs multi-view graph representations to capture diverse linguistic and contextual features, and (2) integrates consensus graph learning with spectral clustering, followed by exemplar-guided LLM topic naming to extract coherent thematic structures, (3) evaluates public opinion through consensus voting among multiple LLMs, and (4) explores temporal dynamics in topic evolution and emotional trends. By integrating these methodological innovations, the research provides a comprehensive, data-driven understanding of public cognition, sentiment, and discourse patterns regarding portable power bank products.

The findings are expected to inform industry design optimization, market strategy refinement, and policy formulation for energy product safety and sustainability. The remainder of this paper is structured as follows: Section 2 describes the data sources and methodology; Section 3 presents the experimental results and analytical findings; Section 4 discusses the results, compares them with previous studies, and highlights the practical implications; and Section 5 concludes with the theoretical contributions, limitations, and directions for future research.

## 2 Materials and methods

**Fig 1:**
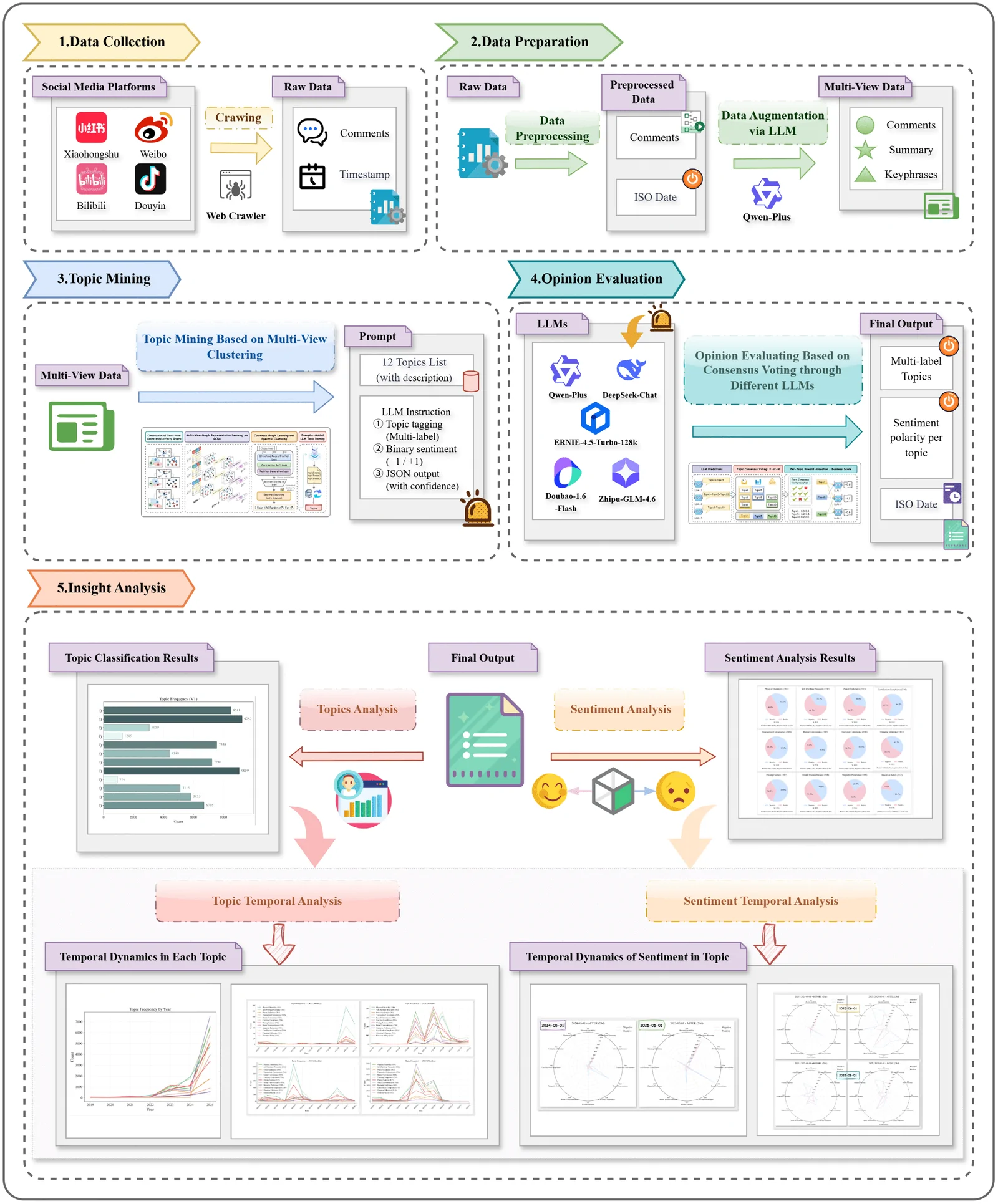
The research process and implementing step.

This study developed an integrated LLMs-driven social-media opinion analysis framework to systematically examine public perceptions of portable power banks in China. The methodological process consisted of five main phases, as shown in Fig 1.

### Data collection

In this stage, public discussion data are collected from major Chinese social media platforms (Douyin, Weibo, Xiaohongshu, Bilibili) using an open-source crawler to obtain topic-relevant comments and timestamps, forming the initial text corpus for subsequent processing (Section 2.1).

### Data preparation

In this stage, the raw corpus is processed through Data preprocessing (Section 2.2.1) and Data augmentation via LLM (Section 2.2.2), transforming raw comments into multi-view textual data (comment, summary, keyphrases) for topic mining.

### Topic mining

In this stage, topic mining is conducted through Topic Mining Based on Multi-View clustering (Section 2.3). Multi-view textual data are processed via Intra-View graph construction and GCN-Based representation learning (Section 2.3.1), followed by Consensus graph learning and spectral clustering (Section 2.3.2). In Exemplar-guided LLM topic naming (Section 2.3.3), representative samples are used to generate topic names and descriptions, and through manual validation, the results are refined into standardized topic definitions. These topic definitions are then formatted into a prompt template, which serves as the input for the next stage, Opinion evaluation.

### Opinion evaluation

In this stage, Opinion evaluating based on consensus voting through different LLMs is performed (Section 2.4). Using the prompt and topic definitions from the previous stage, each comment is evaluated by multiple LLMs to obtain Multi-label Topics and Sentiment Polarity per Topic. A consensus voting mechanism aggregates the outputs into a final structured Opinion, which is exported together with the timestamp (ISO Date) for the subsequent Insight analysis stage.

### Insight analysis

As the final stage, Insight Analysis is conducted using the structured outputs from Opinion evaluation, examining topic patterns, sentiment tendencies, and temporal dynamics to reveal shifts in public attention and emotions (Section 3).

### 2.1 Data sources

According to publicly released statistics by QuestMobile, the total number of unique active users across China’s new media platforms reached 1.071 billion in October 2024, representing an 85.7% penetration rate. Meanwhile, the population of high-value users was estimated at around 238 million. Douyin, Weibo, Xiaohongshu, and Bilibili respectively reported approximately 165 million, 156 million, 88 million, and 70 million high-value users [27]. These platforms possess a large and diverse user base as well as a rich content ecosystem, providing strong representativeness of public opinion and high observability across different contexts. In contrast to e-commerce platforms, where user feedback is typically generated post-transaction and within relatively isolated contexts, comments on social media often arise from dynamic, real-life situations and public discourse, with discussion topics extending beyond purchasing experiences to user needs, policy environments, and overall user experience. Social media comments therefore offer a richer and more multidimensional basis for comprehensively mapping public perceptions of portable power banks.

This study employed a Python-based workflow for data crawling and processing to collect 186,237 publicly available comments related to portable power banks from four major Chinese social media platforms, namely Douyin, Weibo, Xiaohongshu, and Bilibili. After topic-relevance filtering, deduplication and quality inspection, a total of 47,023 valid samples were retained for analysis. The dataset spans from December 2019 to September 2025, covering almost six years of public discourse on portable power banks on major Chinese social media platforms. To reduce potential structural bias associated with platform algorithms, temporal sentiment fluctuations, and echo chamber effects, the final dataset was further organized using time- and platform-based stratification together with text quality control.

All data used in this study were obtained exclusively from publicly accessible content on the target platforms. Data collection and analysis were conducted in accordance with the Terms of Service, Privacy Policies, and public data access rules of each target platform, as well as relevant Chinese legal and regulatory requirements governing data collection and privacy protection. In terms of privacy preservation, only comment text and posting date were retained for subsequent analysis; no personally identifiable information was collected or stored at any stage, including usernames, profile URLs, avatars, device information, IP addresses, or geolocation data. The retained temporal data were restricted to date-level granularity, thereby precluding any identification or tracing of individual users.

### 2.2 Data preparation

#### 2.2.1 Data preprocessing.

This section presents the data preprocessing procedure. Because subsequent stages rely on an LLM to summarize the semantics and key phrases of individual comments (Section 2.2.2), this step prioritizes light cleaning over extensive normalization.

The experiment first removes advertisements and other irrelevant content using regular expressions and random sampling. The remaining comments are subsequently subjected to character-level, non-invasive normalization, which standardizes whitespace and special characters, converts emoticons, reduces noise, and unifies encoding. To preserve linguistic integrity, the process deliberately avoids word- and sentence-level truncation.

Timestamps are unified into the ISO 8601 calendar date (extended format, YYYY–MM–DD), followed by missing-value detection and constraint validation on metadata fields. These operations ensure data quality and structural consistency while preserving colloquial expressions and emotional cues, thereby minimizing adverse effects on semantic modeling.

#### 2.2.2 Data augmentation via LLM.

Short texts on social media often contain substantial noise, limited contextual information, and highly diverse linguistic expressions, leading to sparse semantic spaces and blurred topic boundaries. As a result, traditional embedding models struggle to capture consistent and robust semantic structures [28]. To address this issue, moderate data augmentation can enhance the representation quality of short comment texts.

Supplementary information generated by large language models (LLMs) effectively enhances multiple downstream tasks [29–32]. Viswanathan et al. incorporated LLM-generated keywords before the clustering process, modeling them in parallel with the original representations [33]. The results showed improvements in both semantic consistency and cluster separability, providing empirical support for LLM-based pre-clustering data augmentation.

Based on the above description, this study employs the large language model Qwen3-Plus [34] to generate a summary and keyphrases for each comment prior to clustering. The summary view condenses the main text and reduces noise, while the keyphrase view provides sparse semantic cues to improve interpretability. The comment, summary, and keyphrases are then jointly encoded and normalized using the bilingual embedding model BGE-M3 [35], forming a consistent and robust foundation for multi-view clustering.

### 2.3 Topic mining based on multi-view clustering

Clustering is a feasible approach for unsupervised topic discovery. In short-text and multi-source scenarios, the combination of semantic embedding and similarity learning can effectively capture semantic relationships between texts, resulting in stable and well-separated clusters [36–39]. Therefore, clustering is adopted in this study as the fundamental strategy for topic mining.

This study emphasizes the principle that model architecture should serve data representation. To fully capture the feature information of the augmented texts (Section 2.2.2) across different semantic levels, several well-established mechanisms are integrated into a unified multi-view clustering framework, achieving structural and semantic consistency across views. Specifically, inspired by the SERIES model proposed by Wang and Feng [40], the framework introduces a soft negative-aware contrastive learning mechanism that adaptively reweights negative samples to enhance cross-view semantic alignment.

In addition, the framework employs an edge-level Binary Cross-Entropy (BCE) reconstruction loss with negative sampling to capture intra-view structural dependencies. This paradigm has been widely adopted in recent studies on graph autoencoders and link prediction, such as the standardized link-prediction pipeline in the Open Graph Benchmark [41], diffusion-based negative sampling models [42], large-scale GNN benchmarking for link prediction [43], and subsequent surveys on temporal link prediction [44], among others.

Meanwhile, a Frobenius-norm-based cross-view relational consistency constraint is applied to promote structural alignment, and spectral clustering is performed on the consensus graph to obtain topic partitions. To address the semantic sparsity and cross-view heterogeneity inherent in social media short texts (Section 2.1), an encoder-only multi-view semantic modeling architecture is constructed, which integrates three key objectives, namely structural reconstruction, soft negative-aware contrastive learning, and cross-view relation generation. This design maintains cluster separability while controlling computational and memory costs, thereby enabling stable and interpretable topic discovery.

**Fig 2:**
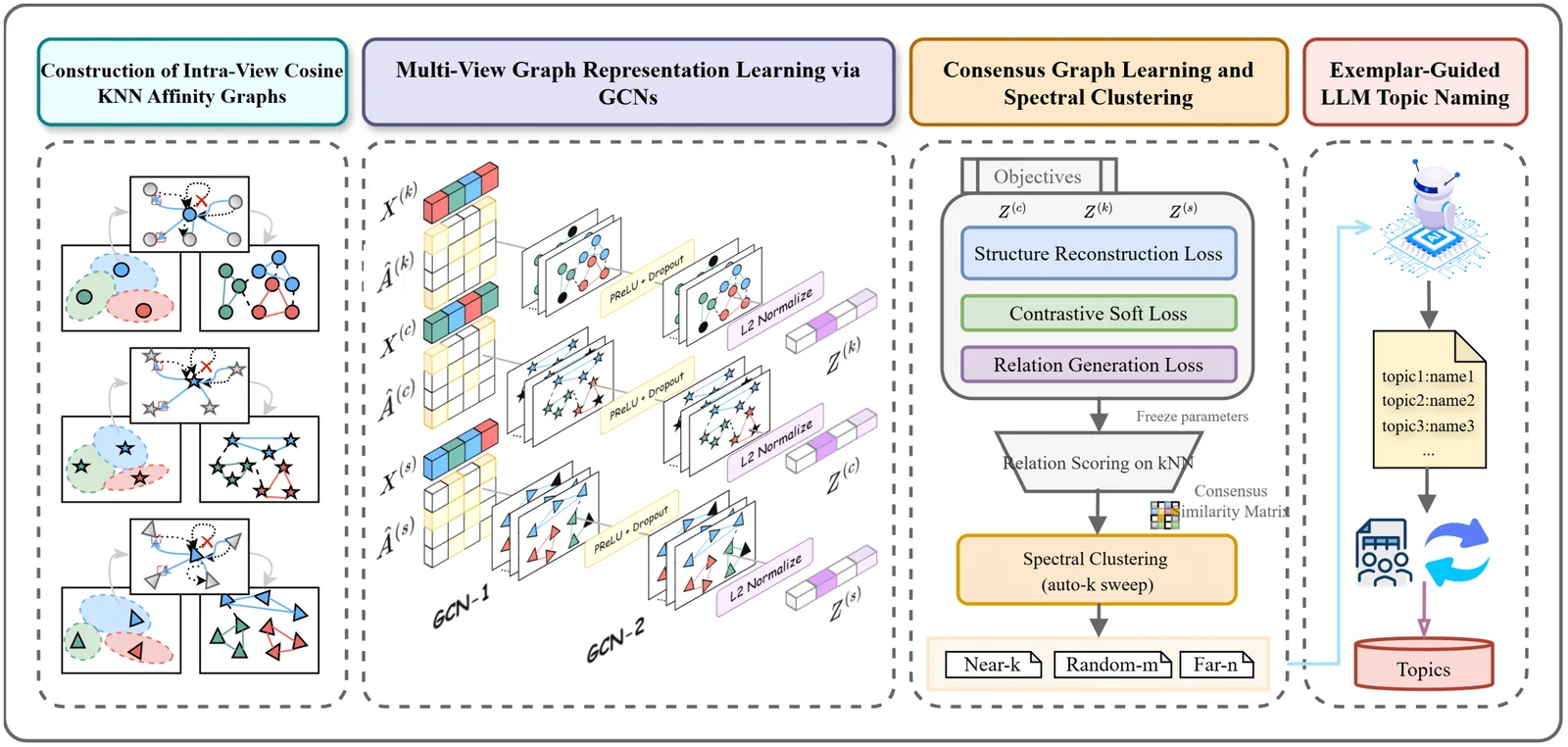
Architecture of the proposed multi-view clustering framework. The framework comprises four stages: (1) construction of intra-view cosine *k*-nearest neighbor (KNN) affinity graphs; (2) multi-view graph representation learning via graph convolutional networks (GCNs); (3) consensus graph learning and spectral clustering; and (4) exemplar-guided large language model (LLM) topic naming.

The overall workflow of the proposed framework is illustrated in Fig 2, which consists of four main stages: (1) construction of intra-view cosine *k*-NN affinity graphs (Section 2.3.1); (2) multi-view graph representation learning via GCNs (Section 2.3.1); (3) consensus graph learning and spectral clustering (Section 2.3.2); and (4) exemplar-guided LLM topic naming (Section 2.3.3).

#### 2.3.1 Intra-view graph construction and GCN-based representation learning.

For the three views (comment, summary, and keyphrases), the normalized semantic representations (Section 2.2.2) serve as inputs to construct intra-view cosine *k*- NN affinity graphs [45,46]. Specifically, within each view, a sparse similarity matrix is generated by retrieving the *k* nearest neighbors of each sample under cosine similarity [45]. The one-directional *k*- NN graph is then symmetrized by the max rule (taking the larger value for each bidirectional pair) and row-normalized to yield a stable random-walk affinity matrix *W*^(*v*)^. Before feeding into the graph network, the affinity matrix is augmented with self-loops and symmetrically normalized to obtain the normalized adjacency matrix ${\hat{A}}^{\left(v\right)}$ used for message propagation. It is defined as follows (see Eq 1):


$$\begin{array}{c} {\hat{A}}^{\left(v\right)}=D^{-\frac{1}{2}}\left(W^{\left(v\right)}+I\right)D^{-\frac{1}{2}} \end{array}$$ (1)

where *W*^(*v*)^ denotes the matrix of view *v*, *I* is the identity matrix, and *D* is the degree matrix defined as $D_{ii}=\sum_{j}(W_{ij}^{\left(v\right)}+I_{ij})$. The normalized matrix ${\hat{A}}^{\left(v\right)}$ is then converted into a sparse tensor for efficient graph input (the random seed is fixed to 42 for reproducibility).

To adapt to the augmented short-text multi-view data, this experiment prioritizes cluster separability while controlling computational and memory costs. An encoder-only representation learning paradigm is adopted, without introducing content decoders or inner-product graph decoders. Each view employs a two-layer Graph Convolutional Network (GCN) encoder to perform two rounds of message propagation on the normalized adjacency matrix ${\hat{A}}^{\left(v\right)}$, yielding structure-aware representations. The outputs are subsequently L2-normalized to stabilize similarity measurement and contrastive learning. The process is formally expressed in Eq 2, Eq 3, and Eq 4.


$$\begin{array}{c} H^{\left(1\right)}={Dropout}_{0.1}\;\left(PReLU\;\left({\hat{A}}^{\left(v\right)}X^{\left(v\right)}W^{\left(0\right)}\right)\right) \end{array}$$ (2)

$$\begin{array}{c} {\hat{Z}}^{\left(v\right)}={\hat{A}}^{\left(v\right)}H^{\left(1\right)}W^{\left(1\right)} \end{array}$$ (3)

$$\begin{array}{c} Z_{i}^{\left(v\right)}=\frac{{\tilde{Z}}_{i}^{\left(v\right)}}{{\left\|{\tilde{Z}}_{i}^{\left(v\right)}\right\|}_{2}+\varepsilon } \end{array}$$ (4)

where *X*^(*v*)^ denotes the input feature matrix of view *v*; ${\hat{A}}^{\left(v\right)}$ is the normalized adjacency matrix of the corresponding view; *W*^(0)^ and *W*^(1)^ are the learnable weight matrices of the two GCN layers; *H*^(1)^ represents the hidden output of the first layer; ${\tilde{Z}}^{\left(v\right)}$ is the unnormalized structural representation; *Z*^(*v*)^ is the final L2-normalized embedding; and ε is a small constant for numerical stability.

#### 2.3.2 Consensus graph learning and spectral clustering.

To achieve structural consistency and optimize cluster separability across multiple views, this stage takes the multi-view representations *Z*^(*v*)^ encoded by GCNs and their corresponding *k*- NN candidate edges (Section 3.3.1) as inputs for cross-view relation scoring and consensus graph learning. The model jointly optimizes three losses including structure reconstruction, soft contrastive, and relation generation, to preserve structural features, enhance semantic discriminability, and promote cross-view consistency.

- Structure reconstruction loss

To preserve the local structural features of each view, a sampled link reconstruction objective is applied to constrain the structural interpretability of the GCN outputs. It is defined in Eq 5.


$$\begin{array}{c} {ℒ}_{\text{struct}}^{\left(v\right)}=-\frac{1}{\left|\Omega^{\left(v\right)}\right|}\sum_{(i,j)\in \Omega^{\left(v\right)}}\;[A_{ij}^{\left(v\right)}\log\sigma \;(z_{ij}^{\left(v\right)})+(1-A_{ij}^{\left(v\right)})\;\log(1-\sigma \;(z_{ij}^{\left(v\right)}))] \end{array}$$ (5)

where $\Omega^{\left(v\right)}$ denotes the set of sampled positive and negative edges, $A_{ij}^{\left(v\right)}$ is the element of the intra-view adjacency matrix, and σ(·) is the sigmoid function. For notational simplicity, we define $z_{ij}^{\left(v\right)}=Z_{i}^{\left(v\right)}\cdot Z_{j}^{\left(v\right)}$ as the inner product between node *i* and node *j* in view *v*. $Z_{i}^{\left(v\right)}$ represents the embedding vector of node *i*. This loss is used to preserve the structural neighborhood relationships within each view.

- Contrastive soft loss

Following the soft negative-aware contrastive learning proposed in SERIES [40], this module, termed Contrastive Soft Loss (CSL), enhances clustering discriminability and cross-view semantic consistency. A soft contrastive objective with a temperature coefficient τ is defined in Eq 6.


$$\begin{array}{c} {ℒ}_{\text{contrast}}=-\frac{1}{N}\sum_{i=1}^{N}\log\frac{\sum_{v_{1}\neq v_{2}}\exp\left(sim\;\left(Z_{i}^{\left(v_{1}\right)},Z_{i}^{\left(v_{2}\right)}\right)/\tau \right)}{\sum_{v_{1},v_{2}}\sum_{j\neq i}\exp\left(sim\;\left(Z_{i}^{\left(v_{1}\right)},Z_{j}^{\left(v_{2}\right)}\right)/\tau \right)} \end{array}$$ (6)

where sim(·) denotes the cosine similarity, and τ is the temperature parameter.

This loss minimizes the distance between positive pairs (representations of the same node across different views) while maximizing the distance between negative samples, thereby reinforcing consistent semantic and structural features across views.

- Relation generation loss

To further capture cross-view structural interactions, a relation generation constraint is introduced to model inter-view edge relationships. The formulation is shown in Eq 7.


$$\begin{array}{c} {ℒ}_{\text{relation}}=\frac{1}{m(m-1)}\sum_{v_{1}\neq v_{2}}{\left\|S^{\left(v_{1}\right)}-S^{\left(v_{2}\right)}\right\|}_{F}^{2} \end{array}$$ (7)

where *S*^(*v*)^ denotes the similarity matrix computed from *Z*^(*v*)^, and $\|\cdot {\|}_{F}$ represents the Frobenius norm.

This term enforces structural consistency of edge relationships across views and facilitates the generation of a unified consensus graph.

The overall loss function is defined by combining the three sub-objectives described above. The complete formulation is shown in Eq 8.


$$\begin{array}{c} ℒ=\alpha \sum_{v=1}^{m}{ℒ}_{\text{struct}}^{\left(v\right)}+\beta {ℒ}_{\text{contrast}}+\gamma {ℒ}_{\text{relation}} \end{array}$$ (8)

where α, β, and γ are weighting hyperparameters that balance the three objectives of structural preservation, semantic contrast, and cross-view consistency.

After completing loss optimization, the similarity matrices *S*^(*v*)^ from all views are combined through weighted fusion to form the consensus graph $S^{*}$. The formulation is given in Eq 9.


$$\begin{array}{c} S^{*}=w_{\text{series}}\cdot \mathrm{Norm}\;\left(\frac{1}{m}\sum_{v=1}^{m}S^{\left(v\right)}\right)+w_{\text{orig}}\cdot \mathrm{Norm}\;\left(A^{\left(v\right)}\right) \end{array}$$ (9)

where *w*_series_ = 0.7, *w*_orig_ = 0.3, and Norm(·) denotes the normalization operation.

Finally, spectral clustering is performed on the consensus graph $S^{*}$. Within the range k∈[10,28], the optimal number of clusters $k^{*}$ is automatically determined through a sweep process based on a composite evaluation function. The value of *k* that maximizes this composite metric is selected as the optimal choice to determine the final topic partitioning results. The composite evaluation function is defined in Eq 10.


$$\begin{array}{c} J(k)=w_{\text{sil}}\cdot Silhouette(k)+w_{\text{nmi}}\cdot NMI(k) \end{array}$$ (10)

where Silhouette(k) represents the composite measure balancing inter-cluster separation and intra-cluster compactness, and NMI(k) denotes the normalized mutual information–based cluster consistency metric. The weighting coefficients are set as *w*_sil_ = 0.6 and *w*_nmi_ = 0.4.

#### 2.3.3 Exemplar-guided LLM topic naming.

In the topic naming stage, this study focuses on clear and verifiable core discussions rather than averaging the semantics of all cluster members. However, given the diverse and context-dependent nature of social media expressions, semantic boundaries among discussions are often blurred. As a result, using all samples within a cluster for naming may reduce topic specificity and increase the chance of semantic overlap. Instead, a small set of representative samples is selected to capture locally coherent micro-discussions that embody stable viewpoints and serve as reliable anchors for defining topic semantics.

After clustering, each cluster’s representatives comprise the *k*_near_ closest, and *k*_far_ farthest, and *k*_rand_ random samples from the fused embedding *Z*, capturing core, boundary, and diversity. These texts are fed to Qwen3-Plus with task prompts to generate a topic name and brief description. The three views (comment, summary, key phrases) are fused with weights {0.4,0.4,0.2}, and outputs are manually reviewed and standardized to yield consistent topic labels.

### 2.4 Opinion evaluating based on consensus voting through different LLMs

The joint voting fusion method can achieve robust consensus aggregation among multiple model outputs, balancing predictive accuracy and system robustness. It is an effective approach for realizing coordinated collaboration among multi-source models [47–49]. However, recent studies indicate that increasing the number of models or calls does not necessarily improve performance; excessive models may cause diminishing returns or amplify noise, while a smaller, high-quality ensemble often yields the best results [48,50].

To achieve more accurate and consistent multi-label and sentiment identification, this study adopts a Three-Party Consensus Voting (3PC) mechanism designed to automatically assess inter-model consistency and dynamically optimize fusion among multiple LLM outputs. Which enhances the precision and reliability of large-scale data mining, providing a robust foundation for subsequent topic and sentiment analysis.

**Fig 3:**
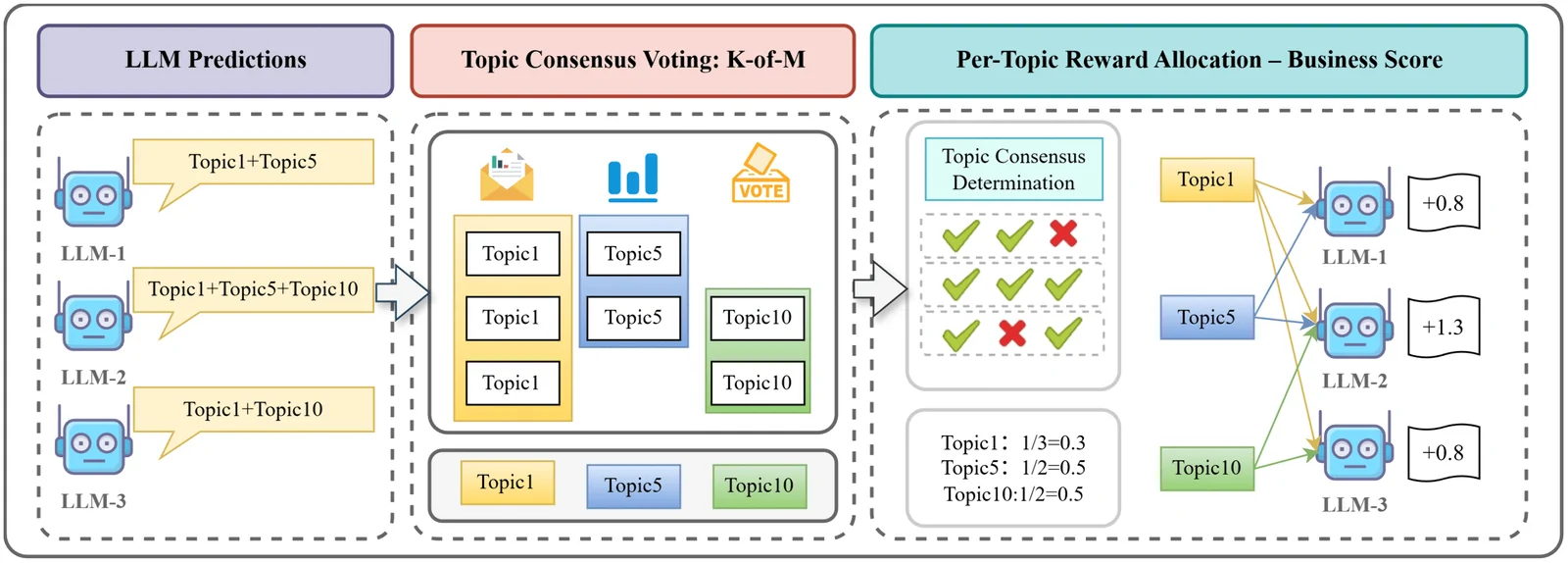
Topic-level consensus and reward allocation in the 3PC mechanism.

The overall process follows a two-level ’topic–sentiment’ structure, performing hierarchical aggregation in fixed, sequential steps. First, the experiment standardizes the output results of all models into a unified structure, filtering out low-quality records based on confidence thresholds to ensure data consistency and reliability. The first stage then determines topic consensus: the system counts all topics proposed by different models, and a particular topic is identified as a consensus topic when it receives support from at least *K* models. If there are multiple candidate topic, they are ranked according to the number of supporting votes, the sum of confidence scores and maximum confidence. The top *N* are then selected as the final results. Fig 3 illustrates this topic-level consensus judgment and reward allocation process.

If no consensus is reached for a sample, the process enters a non-consensus path: the system refers to a model credit ledger to prioritize the currently best-performing model, or, with a certain exploration probability, adopts a cross-model confidence aggregation (max) strategy to balance historical performance utilization and exploration space.

After determining the final topics, the system proceeds to the second-stage sentiment consensus determination. This stage is conceptually aligned with aspect-based sentiment analysis (ABSA), in which sentiment polarity is evaluated with respect to specific aspects rather than the overall text [51,52]. Accordingly, each confirmed topic serves as an aspect unit for polarity voting among its supporting models. When positive or negative polarity meets both the proportion and model-count thresholds, that polarity is accepted as the consensus result; if neither side meets the threshold but valid votes exist, the majority rule is applied. When no valid votes are available from any supporting model, a default polarity is used.

Through this process, every accepted topic obtains a consistent, clear, and stable sentiment judgment. Furthermore, the experiment dynamically updates model performance through two credit ledgers, one for topics and one for sentiments. The credit mechanism follows four rules:

- Fixed distribution: rewards for each accepted topic or sentiment are evenly shared among participating models [53];
- Per-sample cap: prevents excessively high scores for long or complex samples [54];
- Decay and exploration: old credits decay proportionally, while random priority adjustments occur during cold-start and exploration stages [55];
- Over-prediction regularization: models producing too many topic outputs receive proportionally reduced rewards, encouraging precise prediction [56].

This mechanism establishes a self-regulating fusion framework centered on consensus priority, credit-driven weighting and confidence-based assistance. This enables automatic convergence from independent model outputs to stable consensus results. Ultimately, the system generates unified consensus files and credit records for each model ensemble, providing a basis for subsequent performance analysis and collaborative optimization.

## 3 Results and analysis

This section presents and analyzes the structured outputs produced by the methodological workflow (Section 2). First, the final thematic structures are obtained and clustering performance is evaluated based on the multi-view clustering process (Section 2.3). Next, topic assignments and aspect-level sentiment polarity are determined through the consensus voting mechanism across multiple LLMs (Section 2.4), followed by an analysis from two perspectives: topic distribution (Section 3.2.2) and sentiment tendency (Section 3.2.3), revealing public attention and attitude patterns. Finally, the results are extended to the temporal dimension to examine changes in topic prominence and sentiment polarity over time, uncovering dynamic trends in public discussions (Section 3.3). Together, these results constitute the complete insight analysis of this study (Section 2).

### 3.1 Results of topic mining with multi-view clustering

This subsection presents the results of Topic Mining based on Multi-View Clustering (Section 2.3). It first evaluates the clustering performance to validate the effectiveness of multi-view representation learning (Section 3.1.1), and then reports the final topic structures obtained from the clustering process (Section 3.1.2).

#### 3.1.1 Multi-view clustering performance.

In the topic mining framework developed in this study, multi-view clustering serves as a key intermediate module between representation learning and LLM-based semantic interpretation. It integrates semantic information across multiple views to construct candidate topic structures. In this section, the effectiveness of the proposed method is systematically evaluated from multiple perspectives, as detailed below.

### Clustering quality

After completing multi-view graph encoding and tri-objective optimization, the model converged stably. Spectral clustering on the fused consensus graph selected $k^{*}=12$ within k∈[10,28].The clustering quality is supported by a high Calinski–Harabasz index (11,621), a low Davies–Bouldin index (1.02), and a clear gap between intra- and inter-cluster similarities (0.0553 vs. 0.0149). Cross-view consistency is acceptable, achieving an Normalized Mutual Information (NMI) of approximately 0.42. Taken together, these measurements indicate discernible inter-cluster separation and suggest satisfactory intra-cluster compactness and cross-view agreement, providing a sufficient basis for subsequent topic naming.

### Stability analysis

Following the clustering results above, a repeated subsampling stability analysis was conducted over 30 independent runs, each drawing 80% of the full dataset without replacement. The auto-selected number of topics converged predominantly to 12 in 20 of the 30 runs (66.7%), with the remaining runs selecting adjacent values between 10 and 13 ($\bar{k}=11.6$, SD = 0.77), reflecting a stable and data-driven preference for the 12-topic structure. Structural consistency across runs was further evaluated by computing pairwise Adjusted Rand Index (ARI) and NMI between every pair of independent clustering solutions on their shared samples. Across all 435 run pairs, the mean pairwise ARI reached 0.670 (95% CI [0.662, 0.677]) and NMI reached 0.755 (95% CI [0.751, 0.760]). Given that differing numbers of topics between runs mechanically constrain structural comparability, the 206 pairs in which both runs independently selected the same number of topics were examined separately, where pairwise ARI rose to 0.731 (95% CI [0.724, 0.738]) and NMI to 0.793 (95% CI [0.789, 0.796]). Across the 12 topics, the partition showed relatively high stability under independent data perturbations, and the semantic separation space constructed by the clustering framework remained generally robust, with the latent discourse structure retaining a fairly consistent overall organization.

### Topic interpretability evaluation

Content-level alignment provides a more effective measure of topic validity than automated clustering metrics [57,58]. To evaluate topical appropriateness, i.e., how well a single comment fits its assigned topic, representative samples of three types (nearest, random, and farthest) were drawn from the embedding space, consistent with the topic naming stage.

Each sample was independently rated by three experienced social media users on a five-point Likert scale. For each topic, a weighted appropriateness score was computed to measure the overall quality of LLM-generated topic names, while comparing scores across the three exemplar types served to assess the robustness of topic naming under decreasing representativeness.

In the context of the high subjectivity and semantic ambiguity of social media comments, the manual evaluation still showed acceptable inter-rater agreement, with Krippendorff’s α reaching 0.679 under the ordinal level and 0.702 under the interval level, and Fleiss’ κ reaching 0.427.

The detailed scoring results are presented in Table 2, the average topic appropriateness score of the cluster core samples was 4.55, while the weighted average score across the three sample types was 3.88, indicating that topic naming maintained stable semantic validity even when sample representativeness decreased.

**Table 2:** Evaluation of LLM-named topics based on representative samples.

ID	Topical appropriateness			Weighted average
	Nearest	Random	Farthest	
T01	4.3583	3.6667	2.5333	3.8190
T02	4.8333	4.1333	2.3753	4.1566
T03	4.0083	3.5553	1.7333	3.4237
T04	4.9333	3.7778	2.5778	4.1809
T05	4.9167	3.4433	2.4778	4.0784
T06	4.8917	4.1789	2.4000	4.2050
T07	4.4583	2.8444	1.9989	3.5855
T08	3.7333	3.8676	3.0667	3.6192
T09	5.0000	4.0233	2.5767	4.2714
T10	5.0000	4.8899	1.9333	4.3193
T11	4.4000	3.2000	2.2899	3.6907
T12	4.0083	2.9122	1.4878	3.3233
**Average**	**4.5451**	**3.7077**	**2.2876**	**3.8819**

*Note:* A single domain expert rated topical appropriateness on a 1–5 Likert scale (1 = Not aligned, 2 = Slightly aligned, 3 = Moderately aligned, 4 = Well aligned, 5 = Highly aligned). Samples of three types (nearest, random, farthest) were drawn from the embedding space in an 8:3:3 ratio, and the Weighted average column represents the weighted mean score.

These findings demonstrate that the core samples selected for topic naming exhibit relatively strong semantic cohesion and internal consistency, effectively supporting the rationale of the LLM-based naming process. Furthermore, in the subsequent multi-label propagation across the full dataset, these core topics were consistently manifested on a larger scale (Section 3.2.2), confirming that the established topic framework possesses strong semantic representativeness and coverage capacity, thereby providing a solid foundation for subsequent large-scale topic mining and sentiment analysis.

### Comparative experiments

To evaluate the effectiveness of the adopted multi-stage topic discovery method integrating multi-view clustering and LLMs, we conducted a systematic comparison with five representative multi-view clustering methods, namely MVGRL [59], MCGC [60], MFLVC [61], CMGEC [62], and CoMVC [63]. These methods span diverse technical paradigms, including graph contrastive learning, multi-granularity fusion, feature-complementary modeling, and consistency constraints. All methods were evaluated under the same downstream LLM-based topic naming and evaluation pipeline to ensure a fair and consistent comparison.

The evaluation was conducted from two perspectives, namely topic separation and topic quality. For topic separation, inter-centroid distance (ICD), intra-cluster similarity (Sim_intra_), and inter-cluster similarity (Sim_inter_) were adopted to measure the structural separability of clustering results in the embedding space [64]. Since the ultimate goal of clustering in this study is to support LLM-assisted topic discovery rather than clustering itself, it is also important to assess the quality of the resulting topic set. The above separation metrics assess structural properties in the embedding space but do not capture whether the discovered topics are semantically distinct at the naming level. To this end, we further introduced two diversity metrics defined over the final topic set. Intra-List Similarity (ILS) [65] measures the average semantic similarity among topic names within the topic set. A lower ILS indicates that the discovered topics are less semantically redundant and therefore more distinguishable from one another. We also employed the Vendi Score (VS) [66], a similarity-based diversity metric that quantifies the effective diversity of a set based on the eigenvalue distribution of its similarity matrix. A higher VS suggests that the final topic set contains a larger number of effectively distinct semantic directions. These two metrics capture different aspects of the final topic set: the former reflects the degree of semantic proximity among topics, whereas the latter characterizes the effective diversity embodied by the topic set as a whole, thereby providing a more comprehensive complementary evaluation.

The comparative results, evaluated on the representative exemplar subset composed of nearest and randomly sampled instances from each cluster, are presented in Table 3. Overall, all comments concern multiple aspects of usage experience and related concerns surrounding a single product category, namely power banks. Accordingly, the metric distributions across methods reflect the typical characteristics of short-text topic mining in a single-domain setting and fall within an interpretable and reasonable range. Against this background, our method shows a relatively stable overall profile. In particular, it achieves favorable results in both topic-center separation and intra-cluster semantic consistency, with Sim_intra_ reaching 0.7418, indicating that the resulting clusters are internally more coherent. This is important for the present study, because multi-view clustering primarily serves to provide a stable upstream grouping basis for subsequent LLM-assisted topic naming.

**Table 3:** Comparative results of multi-view clustering methods on topic quality metrics.

Method	*k*	Topic Separation			Topic-set Diversity	
		ICD ↑	Sim_intra_ ↑	Sim_inter_ ↓	ILS ↓	VS ↑
MVGRL	12	0.0606	0.6643	0.6295	0.6154	4.1740
MCGC	14	0.0609	0.6463	0.6126	0.5985	4.4269
MFLVC	12	0.0826	0.6714	0.6214	0.6019	4.2415
CMGEC	10	0.1072	0.6688	0.6022	0.5224	4.6185
CoMVC	10	0.1343	0.6816	**0.5949**	0.4898	6.0923
Ours	12	**0.1346**	**0.7418**	0.6447	**0.4732**	**6.2117**

*Note: k* denotes the number of clusters. For baseline methods, *k* was set to the reported or selected configuration; for our method, *k* was determined by the silhouette-based sweep described in Section 2.3.2. All embeddings were computed using BGE-M3 dense vectors. Bold indicates the best value in each column; ↑ and ↓ denote that higher and lower values are preferred, respectively.

On this basis, our method also achieves favorable ILS and VS results, suggesting that the final topic-label set supported by this cluster structure exhibits lower semantic redundancy and higher effective diversity. Although Sim_inter_ is not the lowest among the compared methods, this pattern can be understood in light of the method’s tendency to place greater emphasis on intra-cluster convergence through contrastive learning and structure reconstruction. Under this clustering tendency, some degree of cross-cluster semantic proximity is still retained at the embedding level. At the same time, the final topic-label set continues to show comparatively good ILS and VS results, suggesting that this embedding-level proximity does not directly carry over to the quality of the resulting topic labels.

Taken together, the clustering module adopted in this study showed a balanced overall performance across the evaluated metrics, providing candidate topic structures with favorable discriminability and low redundancy for subsequent downstream tasks.

#### 3.1.2 Results of topic mining.

After the clustering stage, topic names and descriptions were refined through an expert validation process. The initial topic labels were automatically generated by the Qwen-3 large language model, which summarized each cluster based on representative samples, extracted key phrases, and semantic reasoning prompts. To ensure semantic accuracy and interpretability, the model-generated names and justifications were subsequently reviewed and consolidated by three domain experts with back-grounds in social media content analysis, consumer behavior research, and semantic annotation. Each expert independently examined the generated outputs and sample texts, then resolved disagreements through group discussion to produce the final unified topic set. The resulting taxonomy comprises 12 topics, each representing a distinct dimension of public perception toward portable power banks. Serving as the semantic foundation for subsequent multi-label classification and sentiment analysis, this taxonomy ensures methodological consistency and interpretability across the analytical workflow, as summarized in Table 4.

**Table 4:** Finalized topic names and descriptions.

ID	Topic Name	Description
T01	Physical Durability	Concerns the physical robustness and longevity of power banks, including structural integrity and resistance to wear. It reflects user experiences with issues such as loose ports, button failures, fast discharge, or frequent repairs, excluding electrical safety incidents.
T02	Self-Purchase Necessity	Captures attitudes toward the necessity of owning or carrying a personal power bank. It includes perceptions of convenience, frequency of use during travel, and comparisons between self-purchase and rental options.
T03	Power Endurance	Represents evaluations of battery duration and energy retention, focusing on the number of full charges provided, discharge performance, standby power loss, and capacity degradation over time.
T04	Transaction Convenience	Describes the ease and efficiency of purchase, return, and after-sales processes, encompassing logistics, refund procedures, and customer service responsiveness.
T05	Rental Convenience	Describes the ease and flexibility of renting and returning power banks through rental services, focusing on location availability, time accessibility, and overall user experience with the borrowing process.
T06	Carrying Compliance	Describes the practicality of carrying power banks during travel, encompassing both external restrictions such as transportation or venue policies and physical portability factors like weight, size, and design that affect ease of carrying.
T07	Pricing Fairness	Reflects perceptions of the rationality and fairness of power bank prices or rental fees, covering evaluations of affordability, promotions, and perceived cost-performance balance.
T08	Brand Trustworthiness	Focuses on brand-level perceptions and predispositions, describing how brand identity and reputation shape overall evaluation and choice of power banks.
T09	Magnetic Preference	Focuses on the need for and experience with the magnetic attachment function of power banks, covering attachment strength and alignment, stability during movement, compatibility with cases and accessories, and perceived effectiveness of magnetic charging in everyday use.
T10	Certification Compliance	Describes the conformity of power banks with required official certifications and labeling standards, focusing on the authenticity, completeness, and visibility of safety marks such as 3C or equivalent regulatory identifiers.
T11	Charging Efficiency	Represents assessments of charging performance, including speed, compatibility with fast-charging protocols (e.g., PD), power delivery accuracy, and cross-device or cable efficiency.
T12	Electrical Safety	incident reports (overheating, swelling, leakage, smoke, fire/explosion) and confidence in protections (short/over-current safeguards, thermal), considering risks or assurances from typical use, cables, and device compatibility.

### 3.2 Results of opinion evaluating with LLMs consensus voting

#### 3.2.1 Voting performance in different LLMs.

This section employs five LLMs commonly used for Chinese language tasks, including DeepSeek-Chat, Doubao-1.6-Flash, ERNIE-4.5-Turbo-128k, Qwen-Plus, and Zhipu-GLM-4.6. Randomly sampled data were used for both labeling and evaluation to ensure representativeness and consistency across models.

Before the formal evaluation, we first examined the prediction consistency among the five base LLMs. For topic identification, among 47,023 samples, 80.96% received consistent judgments from at least three models, 54.23% from four models, and 32.55% from all five models. For aspect-level sentiment analysis, polarity was compared only on jointly identified topics. Among 80,922 aspect instances, 82.77% achieved complete polarity agreement, and 73.79% of samples showed complete agreement across all evaluable topics. These results indicate a relatively strong consensus among models, supporting the subsequent use of multi-model voting.

Given the relatively strong consensus observed above, as summarized in Table 5, the proposed voting mechanism significantly improves performance over the single-model baselines, with V1 (DS + ERNIE + QWEN) achieving the best overall scores. The V1 ensemble consists of DeepSeek-Chat (DS), ER-NIE-4.5-Turbo-128k (ERNIE), and Qwen-Plus (QWEN).

**Table 5:** Results of multi-LLMs voting performance.

Model / Combination	Multi-Label topics			Sentiment classification			
	Micro-F1	Macro-F1	Subset Acc	Accuracy	Precision	Recall	F1-score
DeepSeek-Chat (DS)	0.8682	0.8663	0.6642	0.9563	0.9590	0.9349	0.9468
Doubao-1.6-Flash (DOUBAO)	0.8347	0.8141	0.6892	0.8850	0.9558	0.7570	0.8449
ERNIE-4.5-Turbo-128k (ERNIE)	0.8511	0.8351	0.7017	0.9446	0.9070	**0.9664**	0.9357
Qwen-Plus (QWEN)	0.8825	0.8685	0.7608	0.9540	0.9377	0.9536	0.9456
Zhipu-GLM-4.6 (GLM)	0.8272	0.8081	0.6250	0.9253	0.9391	0.8766	0.9068
DS+ERNIE+QWEN (V1)	**0.9559**	**0.9278**	**0.9225**	**0.9787**	0.9718	0.9615	**0.9666**
DS+ERNIE+DOUBAO (V2)	0.9071	0.8887	0.8000	0.9603	**0.9726**	0.9321	0.9519
DS+DOUBAO+QWEN (V3)	0.9306	0.9191	0.8442	0.9604	0.9723	0.9321	0.9517
DOUBAO+ERNIE+QWEN (V4)	0.9087	0.8991	0.8058	0.9519	0.9392	0.9465	0.9428
DS+ERNIE+GLM (V5)	0.9025	0.8902	0.7725	0.9607	0.9571	0.9483	0.9527
DS+QWEN+GLM (V6)	0.9032	0.8932	0.7717	0.9617	0.9599	0.9487	0.9543
DS+DOUBAO+GLM (V7)	0.8883	0.8814	0.7475	0.9389	0.9654	0.8865	0.9243
ERNIE+QWEN+GLM (V8)	0.9142	0.9000	0.8150	0.9615	0.9526	0.9555	0.9541
ERNIE+DOUBAO+GLM (V9)	0.8872	0.8710	0.7650	0.9322	0.9471	0.8880	0.9166
QWEN+DOUBAO+GLM (V10)	0.8946	0.8811	0.7758	0.9385	0.9481	0.9026	0.9248

*Note:* DS = DeepSeek-Chat; DOUBAO = Doubao-1.6-Flash; ERNIE = ERNIE-4.5-Turbo-128k; QWEN = Qwen-Plus; GLM = Zhipu-GLM-4.6. V1–V10 denote tri-model ensemble combinations. Acc = Accuracy. Micro-F1, Macro-F1, and Subset Acc are metrics for multi-label topic detection, while Accuracy, Precision, Recall, and F1-score are metrics for binary sentiment classification.

Based on the best-performing three-model combination (V1) identified in the previous evaluation, we further examine the output tendency differences among its constituent models (DeepSeek-Chat, ERNIE-4.5-Turbo-128k, and Qwen-Plus) and how the consensus mechanism mitigates them. The three models exhibit divergent topic identification tendencies, with over-report rates ranging from 10.1% to 20.3% and under-report rates from 4.6% to 19.2%. In terms of sentiment polarity, ERNIE-4.5-Turbo-128k displays a positive-leaning tendency (ΔPos. =+2.73 pp, FPR = 7.11%) while DeepSeek-Chat leans slightly negative (ΔPos. = −1.05 pp), reflecting opposing output tendencies across models. The consensus mechanism markedly reduces these divergences, bringing over- and under-report rates down to 2.78% and 2.04%, with a net sentiment bias of only +0.42 pp relative to human annotation, collectively supporting the relative reliability of the consensus-derived outputs for subsequent analysis.

#### 3.2.2 Topic classification results.

The topic classification process assigned each social-media comment to one or more semantic categories according to the multi-view clustering and exemplar-guided LLM naming described in last stage (Section 3.1.2). This yielded twelve major topics (T01–T12) that collectively portray the public’s multidimensional evaluation of portable power banks.

**Fig 4:**
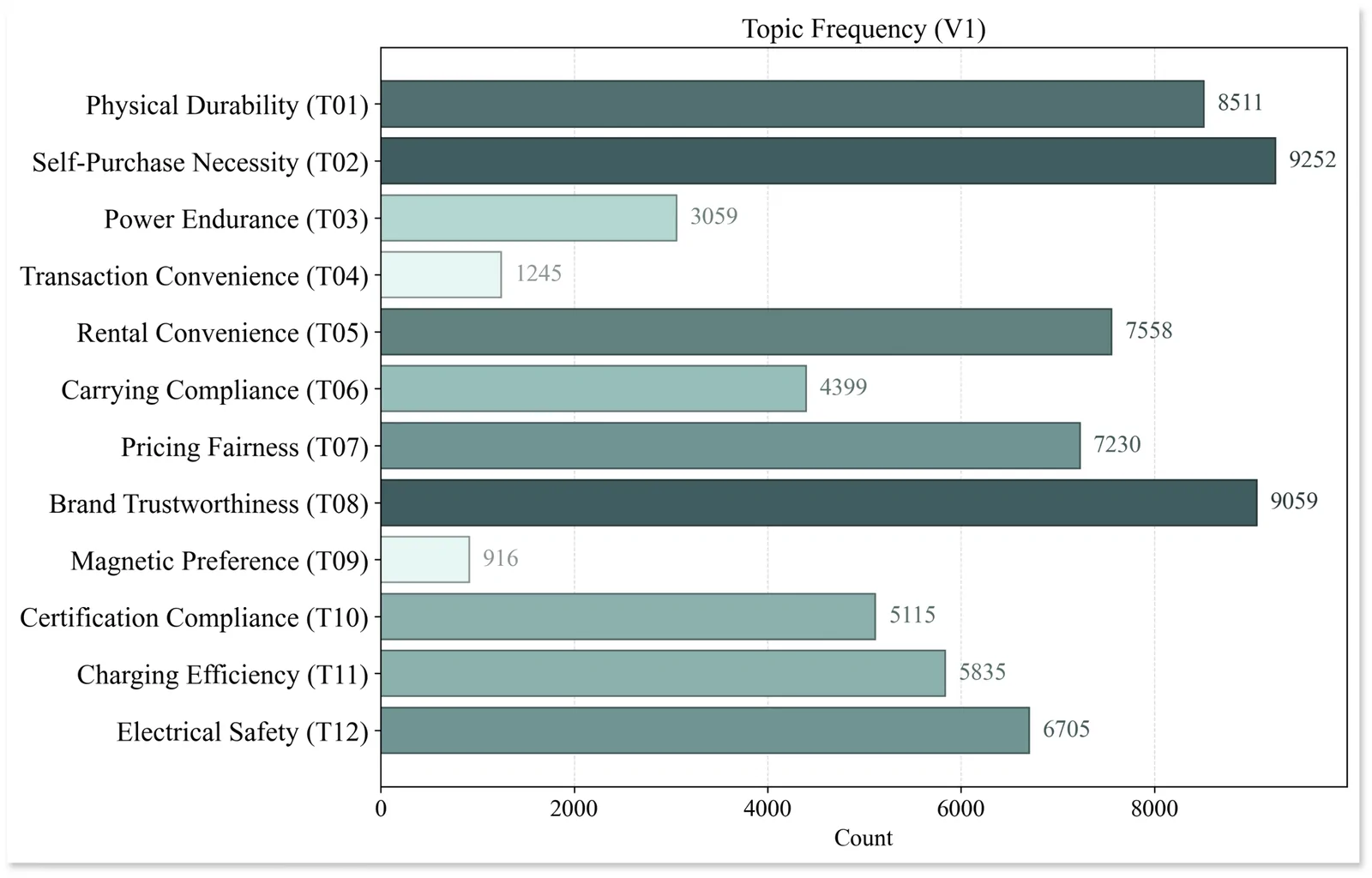
Topic frequency distribution in multi-label classification.

As shown in Fig 4, the classification of 47,023 valid comments resulted in 68,884 topic assignments, with the frequency of each topic corresponding to the number of comments assigned to that topic. Among all annotated entries, “Self-Purchase Necessity” (T02) exhibits the highest occurrence, underscoring that the most salient issue in public discussions is whether individuals should own personal power banks versus relying on shared rental services. This finding highlights the centrality of self-use convenience in shaping user behavior.

The second-most frequent topic, “Brand Trustworthiness” (T08), indicates that consumers place strong emphasis on brand reputation, authenticity, and credibility when evaluating portable power products. Closely following are “Physical Durability” (T01) and “Pricing Fairness” (T07), which together reveal people’s practical concerns about cost–performance balance, material quality, and product longevity. These four topics account for a large share of total mentions and form the core cognitive axis of public discourse. Mid-level frequencies are observed for “Rental Convenience” (T05), “Electrical Safety” (T12), “Charging Efficiency” (T11), and “Certification Compliance” (T10). Their prominence reflects increasing attention to functional reliability and safety assurance in both purchase and rental contexts. “Carrying Compliance” (T06) also appears notably often, suggesting that portability, such as weight, size, and transport restrictions, remains a significant determinant of satisfaction, particularly for travelers.

Conversely, “Transaction Convenience” (T04) and “Magnetic Preference” (T09) occur less frequently. Although representing smaller portions of the corpus, these topics capture context-specific experiences, such as after-sales logistics and emerging magnetic-charging preferences, that nonetheless enrich the thematic landscape. The overall distribution therefore demonstrates a balanced dual structure: functional dimensions (durability, safety, charging performance) co-exist with evaluative dimensions (brand trust, pricing fairness, purchase necessity), together outlining the cognitive profile of public opinion. Moreover, the large-scale outcomes confirm the stability of the topic schema obtained in the pilot stage (Section 3.1.1), validating the robustness of the LLMs-based classification.

**Fig 5:**
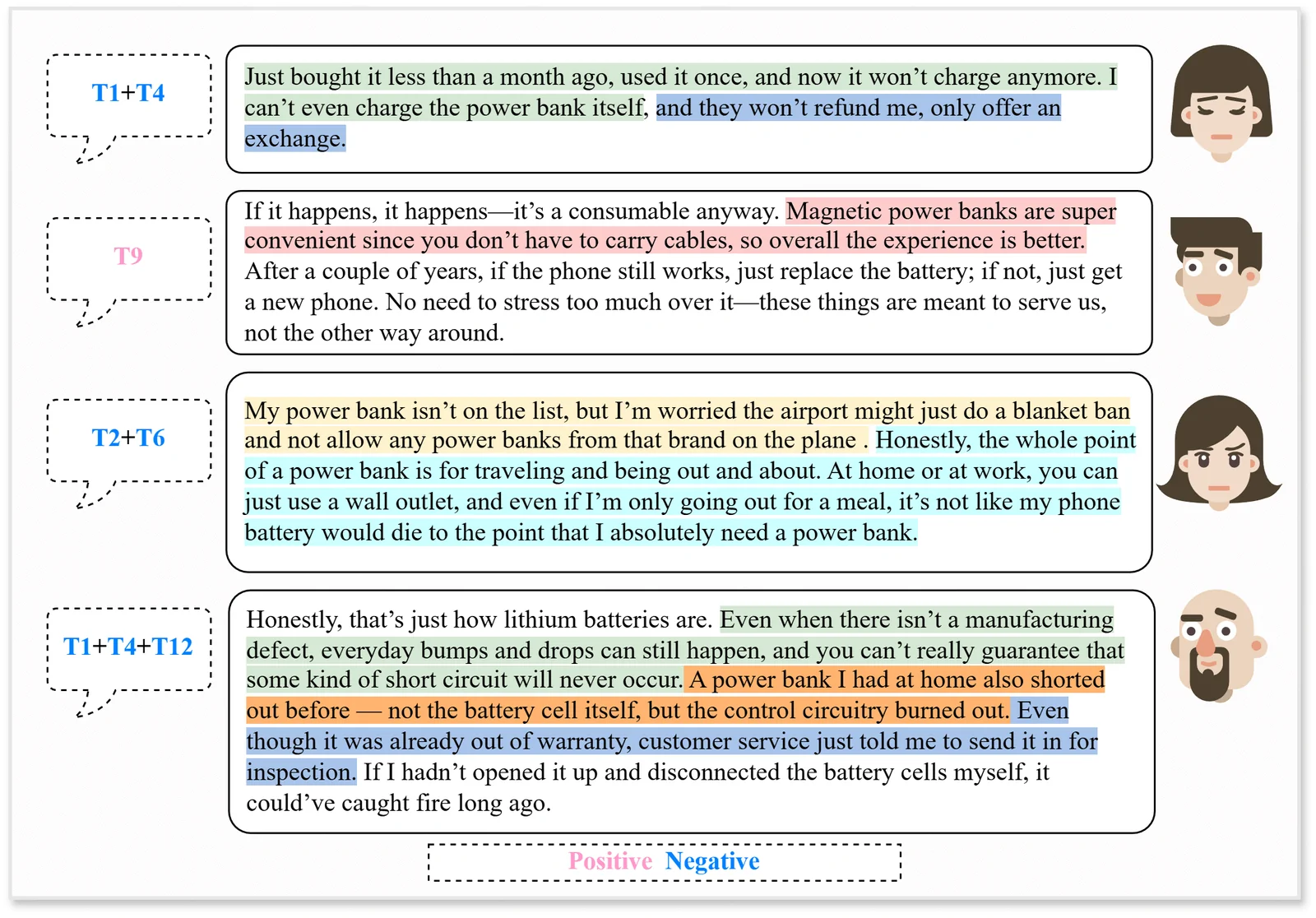
Representative examples of comments.

At the comment level, an average of approximately 1.47 topics were assigned to each comment, with different dimensions of user experience and product perception often appearing together. This pattern is illustrated by representative examples in Fig 5.

Against this background, co-occurrence analysis further reveals how users connect multiple concerns within the same comment. Table 6 summarizes the nine most common topic combinations among 247 unique co-occurrence groups, encompassing 17,182 comments (approximately 36.5% of all valid entries). The results demonstrate that public concerns are interlinked rather than isolated.

**Table 6:** Major co-occurring topic pairs and their proportions.

No	Topic combination	Frequency	Proportion
1	Physical Durability (T01) + Brand Trustworthiness (T08)	1,151	2.45%
2	Rental Convenience (T05) + Pricing Fairness (T07)	937	1.99%
3	Physical Durability (T01) + Electrical Safety (T12)	922	1.96%
4	Carrying Compliance (T06) + Certification Compliance (T10)	888	1.89%
5	Self-Purchase Necessity (T02) + Rental Convenience (T05)	858	1.82%
6	Brand Trustworthiness (T08) + Electrical Safety (T12)	779	1.66%
7	Physical Durability (T01) + Brand Trustworthiness (T08) + Electrical Safety (T12)	620	1.32%
8	Pricing Fairness (T07) + Charging Efficiency (T11)	521	1.11%
9	Pricing Fairness (T07) + Brand Trustworthiness (T08)	490	1.04%
–	–	–	<1% omitted
**Total (247)**	–	**17,182**	**36.54%**

*Note:* The “Total” row summarizes 247 unique co-occurring topic combinations, comprising 17,182 comments (36.54% of all valid entries). Topic pairs with a share below 1% are omitted.

Specifically, the most frequent topic combination is “Physical Durability” (T01) and “Brand Trustworthiness” (T08), which appears in approximately 1,151 comments, accounting for about 2.45% of the corpus, which indicates perceptions of structural reliability are tightly coupled with brand credibility. Consumers often attribute durability failures or successes directly to brand quality assurance, implying that trust in brand acts as a mediating factor between physical performance and purchase confidence.

The next two frequent pairs, “Rental Convenience” (T05) with “Pricing Fairness” (T07) and “Physical Durability” (T01) with “Electrical Safety” (T12), reflect practical trade-offs between service accessibility, cost, and perceived risk. The coupling of “Carrying Compliance” (T06) and “Certification Compliance” (T10) further indicates that public often evaluate physical portability together with official conformity, demonstrating awareness of standardized safety marks during travel use. Other significant associations include “Self-Purchase Necessity” (T02) with “Rental Convenience” (T05), highlighting behavioral comparisons between ownership and shared usage, and “Brand Trustworthiness” (T08) with “Electrical Safety” (T12), revealing that confidence in safety is often anchored in perceptions of brand reliability. Lower-frequency but meaningful clusters, such as “Physical Durability (T01) + Brand Trustworthiness (T08) + Electrical Safety (T12)”, demonstrate multi-topic integration where mechanical reliability, brand image, and safety perception jointly frame public perception.

#### 3.2.3 Sentiment analysis results.

Sentiment analysis indicated significant differences in polarity distributions across the twelve topics ($\chi^{2}(11)=7744.06$, *p* < 0.001), with a moderate association between topic and sentiment (Cramér’s *V* = 0.335). Because the same source comment could contribute sentiment observations to more than one topic, potential within-comment dependence among topic-level observations may affect the interpretation of the significance test. To assess the sensitivity of the observed differences to this potential dependence, a sensitivity analysis was conducted using the 29,841 single-topic comments, representing 63.5% of all comments, with each comment contributing only one observation.

The sensitivity analysis yielded broadly consistent results: differences in sentiment distributions across topics remained statistically significant ($\chi^{2}(11)=3839.40$, *p* < 0.001), with a comparable magnitude of association (Cramér’s *V* = 0.359). The predominant sentiment orientation was consistent for 11 of the 12 topics (91.7%), and the mean absolute difference in positive sentiment proportions between the full-sample and single-topic analyses was 3.7 percentage points.

**Fig 6:**
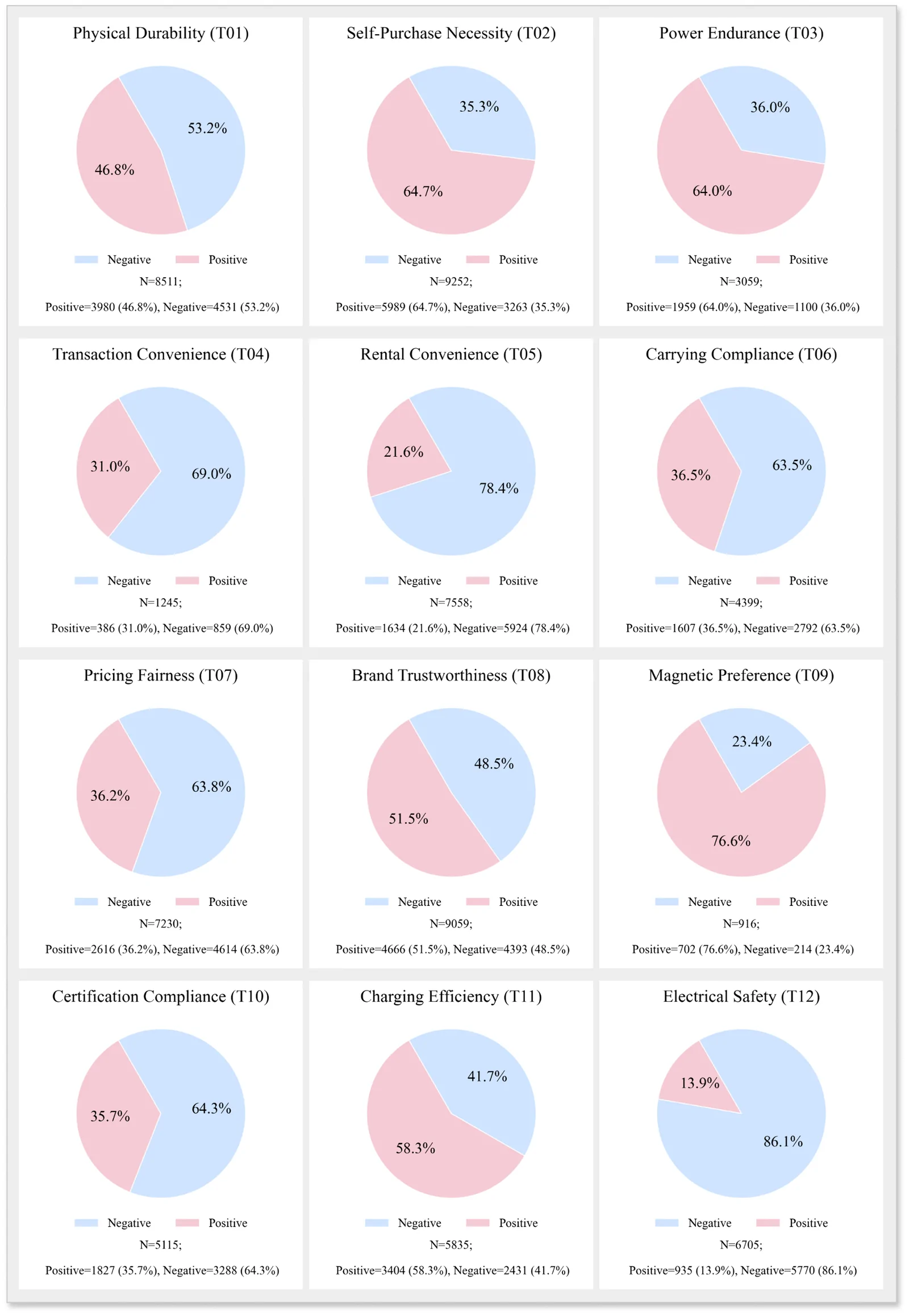
Sentiment polarity distribution by per topic.

Fig 6 visualizes the polarity distribution, with pink representing positive sentiment and blue indicating negative sentiment.

Across the corpus, several topics exhibit markedly skewed sentiment distributions, whereas others show relatively balanced patterns. To quantify the uncertainty surrounding the topic-specific sentiment estimates, Wilson 95% confidence intervals were calculated for the corresponding proportions. Among the most prominent negative dimensions are “Electrical Safety” (T12), “Rental Convenience” (T05), and “Transaction Convenience” (T04). “Electrical Safety” (T12) exhibits the highest proportion of negative sentiment, at 86.1% (95% CI [85.2, 86.9]). Comments in this topic frequently refer to overheating, battery swelling, and explosion risks, as well as concerns about protective mechanisms such as thermal management and short-circuit protection. The pronounced negativity highlights public anxiety regarding product reliability and the perceived insufficiency of existing safety certifications. “Rental Convenience” (T05) follows, with 78.4% (95% CI [77.4, 79.3]) of comments classified as negative, indicating substantial dissatisfaction with shared-use services. Complaints often involve equipment unavailability, delayed maintenance, or inconsistent charging performance across venues. This suggests that, despite the convenience offered by rental models, inconsistent service quality and inadequate service management remain important barriers to public satisfaction. “Transaction Convenience” (T04) ranks third in negative sentiment, at 69.0% (95% CI [66.4, 71.5]), with many people expressing frustration with after-sales logistics, refund delays, and poor responsiveness of customer service systems. These concerns emphasize the importance of efficient consumer protection mechanisms and transparent transaction platforms.

In contrast, “Magnetic Preference” (T09) shows a distinctly positive sentiment orientation, with 76.6% of its sentiment observations classified as positive (95% CI [73.8, 79.3]). The public expresses enthusiasm for the improved attachment stability and ergonomic experience of magnetic charging, associating it with modern design aesthetics and user-friendly innovation. This positive sentiment suggests a strong consumer appetite for emerging energy-interface technologies and reinforces the role of functional novelty in driving product approval.

Several other topics display more mixed or nuanced sentiment patterns. For example, “Brand Trustworthiness” (T08) presents a nearly balanced sentiment distribution (51.5% positive vs. 48.5% negative). People recognize the importance of reputable brands for perceived safety and quality assurance but remain cautious toward marketing claims and counterfeit risks. “Charging Efficiency” (T11), with 58.3% positive sentiment(95% CI [57.1, 59.6]), leans slightly positive, reflecting user appreciation of high-speed charging features, though occasional dissatisfaction arises from cable incompatibility or charging-accuracy deviations. “Physical Durability” (T01), by contrast, tips slightly negative, with 53.2% negative sentiment (95% CI [52.2, 54.3]), suggesting that while users acknowledge solid construction, concerns over rapid wear and port or button failures remain marginally dominant. “Pricing Fairness” (T07) reveals moderate negativity, with 63.8% negative sentiment (95% CI [62.7, 64.9]), reflecting frequent public debate over whether current retail prices or rental fees align with perceived performance value and underscoring persistent sensitivity to cost-benefit ratios. “Power Endurance” (T03) shows a positive majority of 64.0%, whereas “Carrying Compliance” (T06) is predominantly negative at 63.5%, indicating differing evaluations of battery endurance and portability constraints. “Certification Compliance” (T10), though regulatory in nature, shows a noticeable 64.3% (95% CI [63.0, 65.6]) negative sentiment, implying that official marks such as the China Compulsory Certification (3C) labels have yet to instill complete public confidence in product authenticity and safety verification.

Overall, public sentiment toward portable power banks remains measured and context-dependent. While enthusiasm is evident for new features and improved efficiency, concerns about safety, certification, and service quality dominate critical discourse. The coexistence of positive technological optimism and persistent regulatory skepticism underscores the need for both engineering enhancement and stronger governance to reinforce consumer trust in portable energy technologies.

### 3.3 Temporal dynamics of public opinion

Although the temporal distribution of social-media comments displays a strongly event-driven pattern, examining longitudinal trends provides valuable insight into the evolution of public perception. This section analyzes both the macro-level variation across years and the micro-level monthly fluctuations, highlighting how topic salience and emotional tone respond to technological change, market development, and risk events in China’s portable power bank industry.

The analysis proceeds in two parts. Section 3.3.1 explores annual and intra-annual variations in topic attention, capturing long-term structural evolution. Section 3.3.2 (not shown here) further examines sentiment transitions before and after key surges, revealing how positive and negative orientations shift in response to emerging issues.

#### 3.3.1 Temporal dynamics in each topic.

First, this study statistically examined annual changes in the intensity of public discussion using the Mann–Kendall trend test and Sen’s slope, a nonparametric estimator of trend magnitude. Overall, with the exception of “Charging Efficiency” (T11), all topics exhibited significant monotonic increasing trends, indicating that for most topics the null hypothesis of no monotonic trend could be rejected and that the observed changes were unlikely to be explained by random fluctuation. Detailed results are presented in Table 7.

**Table 7:** Annual statistical overview of topic frequency trends.

ID	Topic	Mann–Kendall Test			Sen’s Slope Estimation	
		*Z*	*p*	Trend	Slope	95% CI
T01	Physical Durability	2.10	0.04	↑	254.50	[7.00, 2165.00]
T02	Self-Purchase Necessity	2.40	0.02	↑	436.00	[18.33, 2083.67]
T03	Power Endurance	2.10	0.04	↑	174.25	[2.67, 574.33]
T04	Transaction Convenience	2.40	0.02	↑	61.00	[2.67, 294.00]
T05	Rental Convenience	2.10	0.04	↑	375.40	[60.67, 1504.67]
T06	Carrying Compliance	2.40	0.02	↑	69.50	[5.00, 1318.33]
T07	Pricing Fairness	2.40	0.02	↑	342.00	[17.00, 1683.00]
T08	Brand Trustworthiness	2.40	0.02	↑	206.33	[6.00, 2497.33]
T09	Magnetic Preference	1.97	0.05	↑	59.00	[0.00, 180.67]
T10	Certification Compliance	2.58	0.01	↑	3.00	[1.00, 1696.67]
T11	Charging Efficiency	1.80	0.07	–	311.33	[-4.00, 1100.67]
T12	Electrical Safety	2.40	0.02	↑	144.00	[7.00, 1955.33]

**Fig 7:**
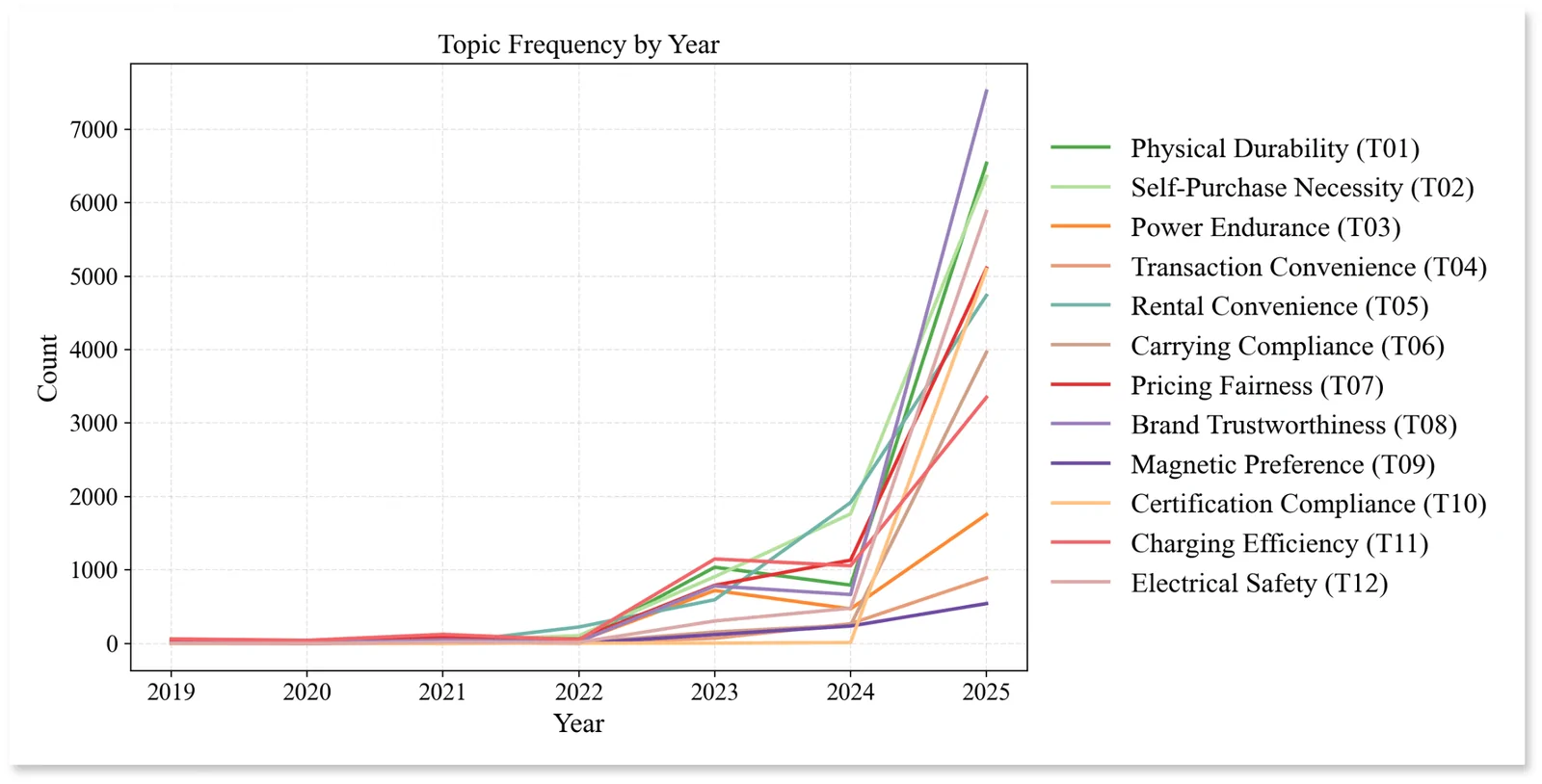
Annual trends in topic frequency.

As shown in Fig 7, from 2019 to 2021, overall discussion volume remained minimal, with discussion largely centered on “Pricing Fairness” (T07), “Charging Efficiency” (T11), and “Power Endurance” (T03)—topics reflecting a performance and value oriented early market phase.

Beginning in 2022, attention expanded markedly. The rise of “Physical Durability” (T01) and “Brand Trustworthiness” (T08) indicates a perceptual shift from purely functional evaluation to more integrated assessments of reliability, safety, and brand credibility. During 2023–2024, this transformation accelerated: topics diversified, and user focus broadened toward experience-, service-, and regulation-related dimensions.

By 2025, nearly all topic curves reach their highest recorded counts, signifying an unprecedented intensification of public engagement. Notably, “Self-Purchase Necessity” (T02) and “Certification Compliance” (T10) surge in tandem with “Brand Trustworthiness” (T08) and “Electrical Safety” (T12), suggesting that product ownership, compliance awareness, and safety concerns have become intertwined. Taken together, the trajectory reflects a maturing discourse: from single-dimensional performance appraisal to a multi-dimensional cognition integrating technological reliability, brand confidence, regulatory trust, and user experience.

**Fig 8:**
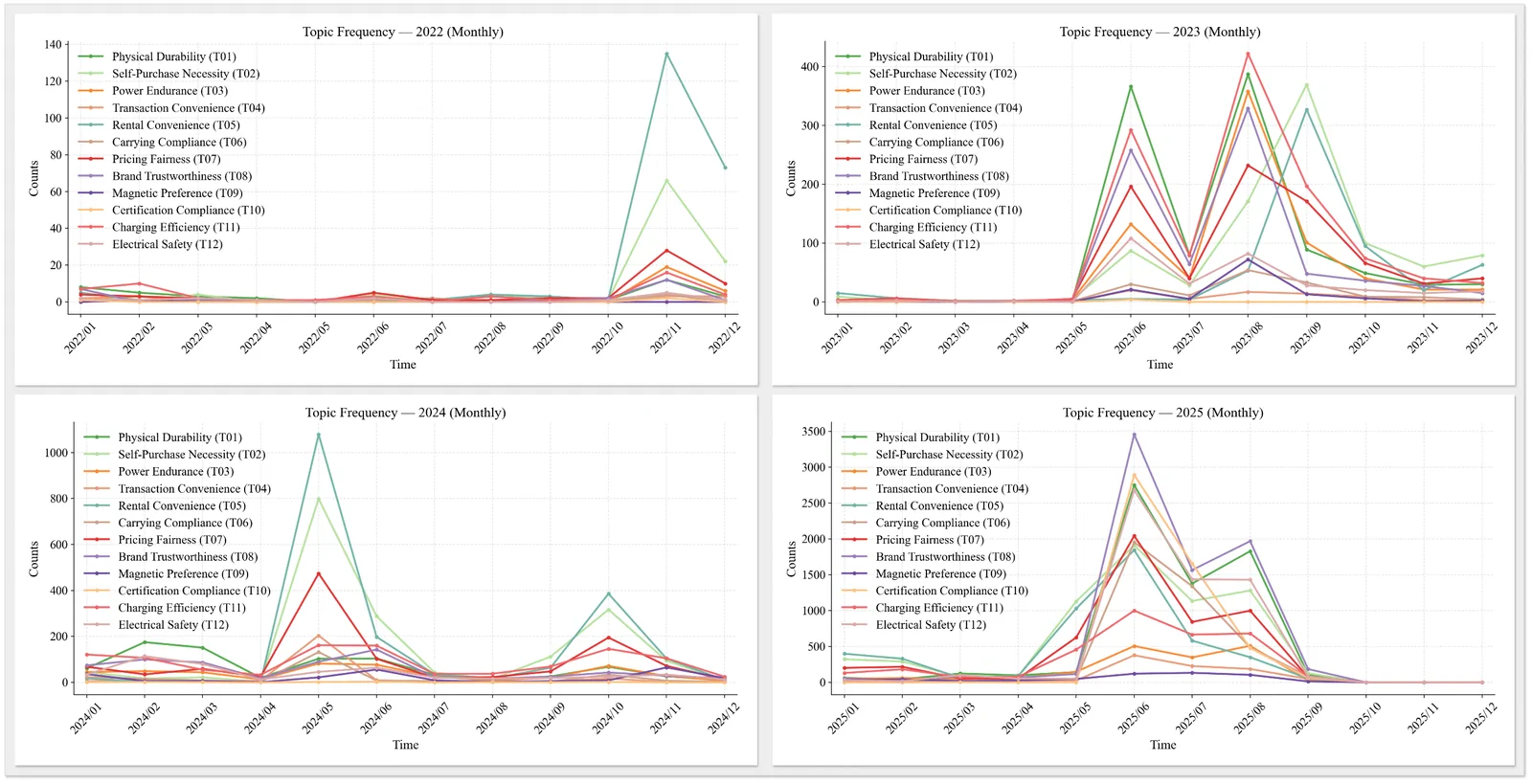
Monthly topic dynamics within each year from 2022 to 2025.

To further unpack the annual patterns described above, Fig 8 details the month-by-month evolution from 2022 to 2025, revealing that comment peaks correspond closely to identifiable industry and societal events.

Discussion intensity remained low throughout most of the year, with a modest rise in November 2022, dominated by “Rental Convenience” (T05) and “Physical Durability” (T01).

Activity expanded substantially, with multiple peaks between June and September 2023. Prominent topics include “Pricing Fairness” (T07), “Brand Trustworthiness” (T08), and “Physical Durability” (T01), often co-occurring. During this period, the discussion showed a clearer event-driven pattern, coinciding with the July 2023 CCC certification announcement and its formal implementation in August (Table 1). Under the influence of these developments, public attention was no longer confined to product performance alone, but increasingly extended to price perception, brand trustworthiness, and product quality.

A single, highly concentrated surge appeared between May and June 2024, when “Self-Purchase Necessity” (T02) and “Rental Convenience” (T05) rose sharply, alongside secondary increases in “Power Endurance” (T03) and “Charging Efficiency” (T11). This pattern may reflect a growing user preference for ownership over rental models, potentially associated with shifts in market supply and consumer purchasing behavior. Discussions during this phase appear to have placed greater emphasis on autonomy and reliability in personal energy access.

The final stage exhibits the highest overall intensity, with synchronized peaks from June to August 2025 across nearly all topics. Particularly, “Brand Trustworthiness” (T08), “Physical Durability” (T01), “Certification Compliance” (T10), and “Electrical Safety” (T12) maintain elevated trajectories, revealing a pronounced re-orientation toward institutional trust and technical assurance. Against the backdrop of the June 2025 safety incidents and subsequent product recalls, as well as the aviation-related restrictions introduced later that month (see Table 1), public discussion was no longer confined to product convenience or pricing, but increasingly turned toward the critical assessment of safety, standards, and corporate credibility.

#### 3.3.2 Temporal dynamics of sentiment in topic.

This section focuses on the discussion surge period from 2024 to 2025 (Section 3.3.1) in order to analysis sentiment across topics and the patterns of its evolution during this phase. Following prior studies indicating that event impacts typically peak within 2–4 weeks, a symmetrical ±4-week (28-day) event window is adopted, excluding the event day [67].

**Fig 9:**
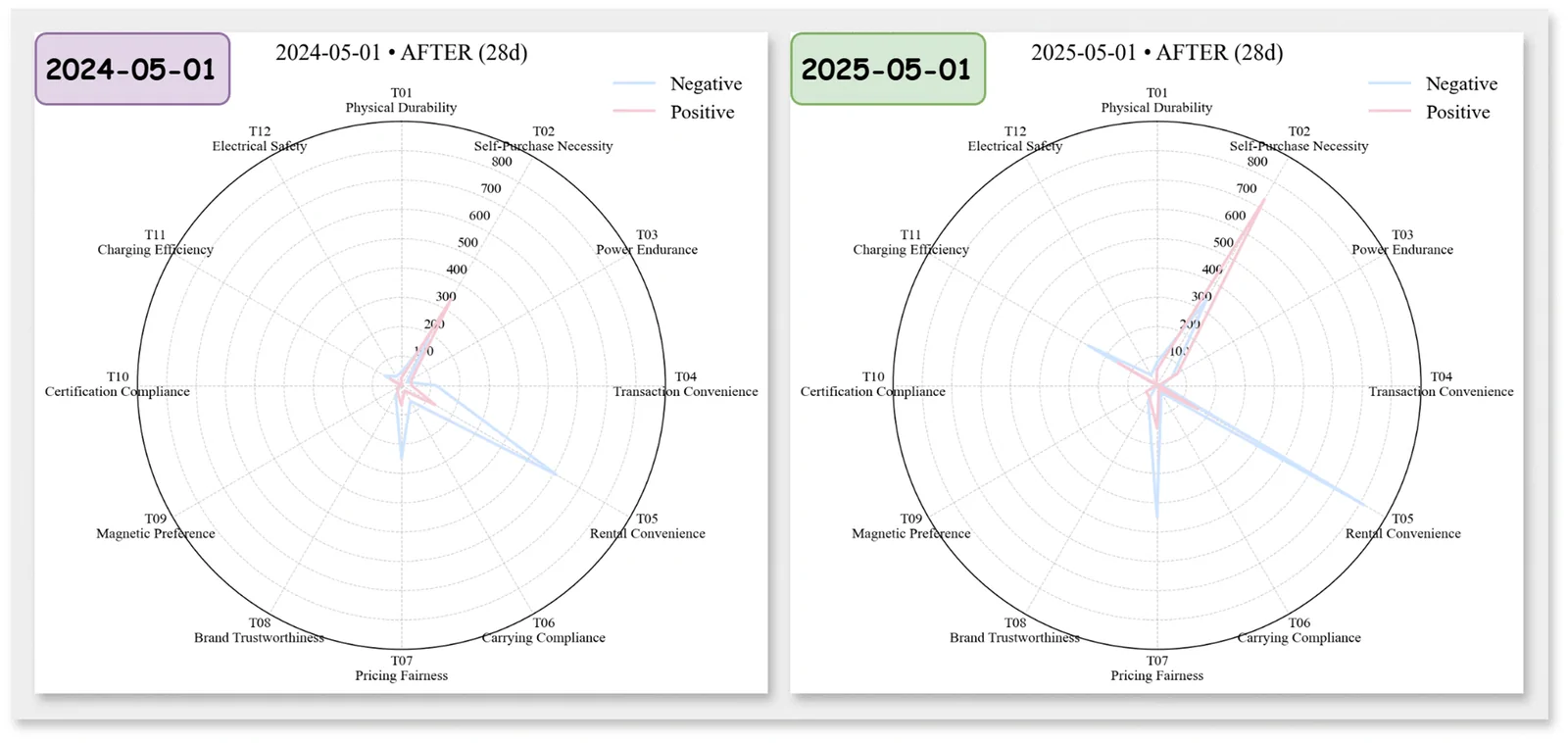
Topic sentiment radar comparison: 2024-05-01 and 2025-05-01 (Post-28 Days).

Fig 9 illustrates the sentiment radar comparison for the 28 days following 1 May in both 2024 and 2025, representing equivalent calendar windows across consecutive years.

In 2024, the post-event sentiment field is relatively compact, dominated by functional and transactional topics. “Self-Purchase Necessity” (T02) remained predominantly positive, consistent with increasing consumer confidence in personal ownership models. Conversely, “Rental Convenience” (T05) and “Pricing Fairness” (T07) retained strong negative polarity, reflecting persistent dissatisfaction with shared-rental reliability and perceived cost imbalance.

By 2025, the sentiment structure exhibits both expansion and differentiation. Overall comment volume increased substantially across nearly all topics, signaling heightened public engagement and diversified emotional expression. Although the general polarity pattern remained stable, two notable shifts occurred: the “Charging Efficiency” (T11) experienced the most pronounced growth in volume, reflecting intensified attention to fast-charging technologies and cross-device compatibility. Despite this surge, negative sentiment still outweighed positive evaluations, underscoring continuing frustration with real-world charging speed and consistency. A mild softening of negativity appeared in “Pricing Fairness” (T07) and “Rental Convenience” (T05), hinting at incremental improvement in public perception of market value and service accessibility.

In a word, the comparison between 2024 and 2025 suggests a transition from narrow functional concern to broader evaluative discourse, as public awareness expanded to encompass both technical performance and service experience dimensions.

**Fig 10:**
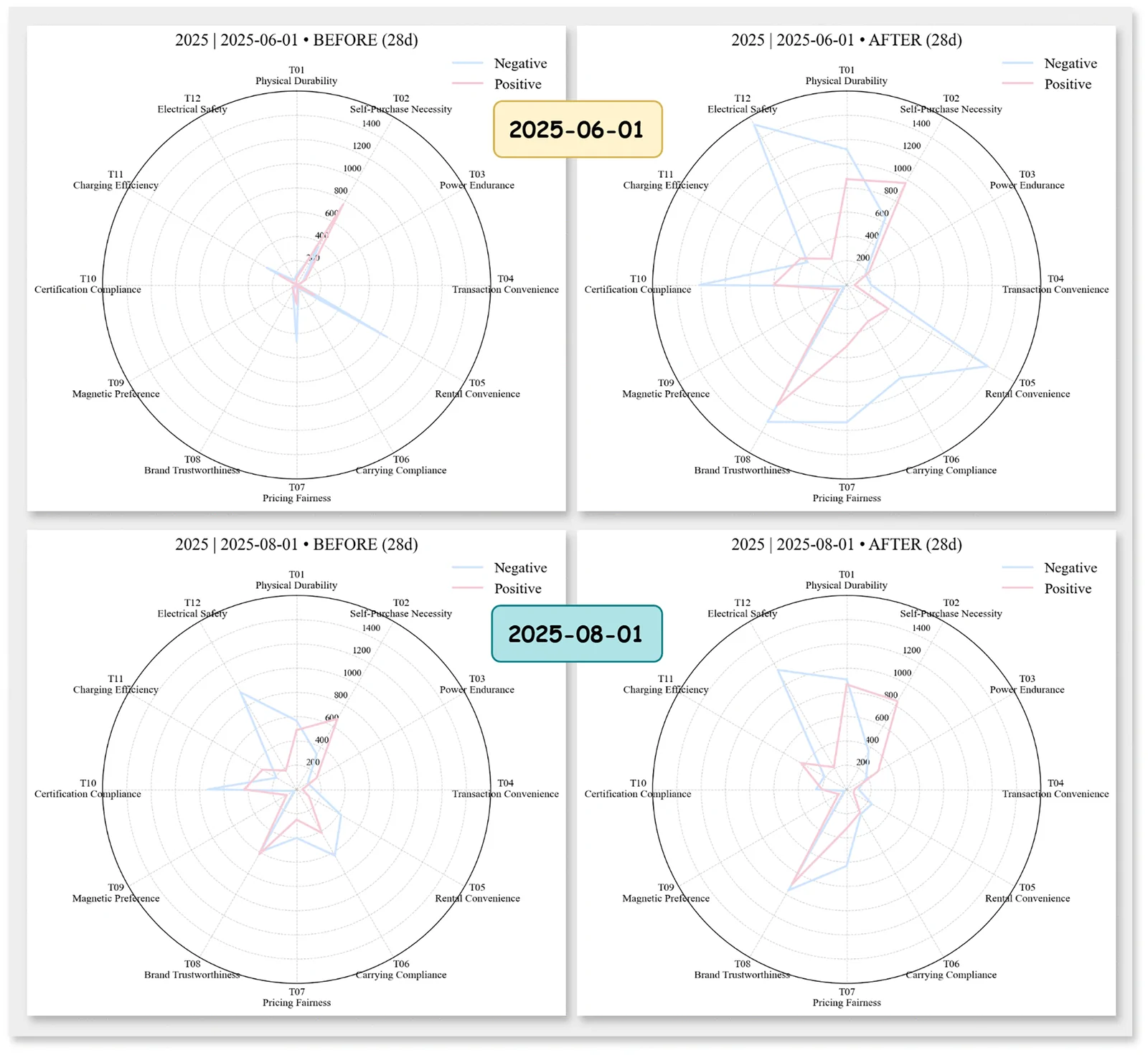
Topic sentiment radar comparison around the two discussion peaks in 2025 (± 28 Days).

Given the substantial growth in discussion scale, 2025 was designated as the core period for detailed intra-year analysis. Two major discussion peaks, which centered on 1 June 2025 and 1 August 2025, were examined using ±28-day symmetric windows to capture sentiment shifts before and after each surge, as show in Fig 10.

The radar plots reveal a clear structural convergence in sentiment distribution following the first peak. Comment volumes expanded across nearly all topics except “Self-Purchase Necessity” (T02), indicating that the event triggered broad discourse extending beyond ownership behavior.

Notably, “Certification Compliance” (T10) and “Charging Efficiency” (T11) underwent the most significant polarity reversals: For “Charging Efficiency” (T11), sentiment shifted toward positivity, suggesting increased optimism regarding new product upgrades or standardization of high-power charging protocols. In contrast, “Certification Compliance” (T10) turned sharply negative, a shift that became more salient after the June 2025 recalls, the suspension of CCC certificates for several brands, and the subsequent aviation-related restrictions (see Table 1). Simultaneously, “Electrical Safety” (T12) and “Carrying Compliance” (T06) saw an expansion of negative sentiment, with public attention increasingly drawn to battery safety risks and the practical constraints introduced by transport-related regulatory measures. Together, these trends highlight an intensification of scrutiny toward safety and compliance dimensions in the wake of the June 2025 safety incidents, recalls, and regulatory actions.

The second surge presents a more stabilized emotional landscape. While overall polarity levels changed modestly, several topic-specific adjustments emerged: Negative mentions of “Certification Compliance” (T10) and “Pricing Fairness” (T07) declined noticeably, suggesting a temporary easing of criticism possibly associated with the partial resolution of the mid-year regulatory events around August 1 (see Table 1), alongside seasonal factors such as summer sales activity. “Brand Trustworthiness” (T08) rose sharply in volume, accompanied by an inversion of sentiment structure: negative opinions slightly exceeded positive ones. This indicates a shift of public focus from technical faults toward brand accountability, as users began to evaluate long-term credibility and service transparency. Positive assessments of “Charging Efficiency” (T11) continued to rise, even as event-related volatility diminished. This persistent positivity implies growing recognition of tangible improvements in fast-charging technology, marking a gradual transition from reactionary complaint to informed endorsement.

## 4 Discussion

This study conducted a comprehensive computational analysis of large-scale social media discourse to elucidate the structure and evolution of public perception and sentiment toward portable power banks in China. By integrating multi-view clustering and LLMs-driven opinion evaluation, the research captures both the topic and sentiment states of public engagement with emerging portable power banks.

### 4.1 Key findings and analytical discussion

The empirical results reveal that public attention consistently centers on product attributes and real-world experience, reflecting stable cognitive priorities over time, despite the increasing technological sophistication of portable power devices, user discourse remains dominated by pragmatic evaluations of durability, efficiency, and price rationality.

Overall, positive sentiment remains prevalent, with positive expressions accounting for roughly two-thirds of the observed sentiment. This sentiment stability across years and event phases (Figs 8–9) suggests that ownership intention and consumer confidence are resistant primarily to short-term external shocks, reflecting a persistently favorable public attitude toward owning power banks.

At the structural level, brand trust and cost-effectiveness emerge as the two most decisive evaluative dimensions. Users repeatedly emphasize price rationality, product lifespan, and reputation credibility as key determinants of satisfaction, indicating a shift from impulsive or convenience-driven consumption toward rational, reliability-oriented decision-making.

Interestingly, the findings also reveal a coexistence of ownership preference and shared accessibility. Although shared power-bank services have proliferated in urban and travel settings, public sentiment toward them is overwhelmingly negative (78–80%), mainly due to dissatisfaction with pricing, device inconsistency, and unreliable maintenance. In contrast, discussions about self-purchased power banks display a markedly positive tone (approximately 65%), underscoring user preference for autonomy, control, and psychological reassurance. Even during high-discussion windows (May 2024 and June–August 2025), this polarity difference persists, indicating a deep-rooted behavioral divergence between the perceived utility of personal versus shared ownership models.

At the same time, topic diversity has expanded beyond core performance evaluations to include safety regulation, transportation compliance, and certification standards. The heightened attention to “Electrical Safety” (T12) and “Certification Compliance” (T10) in 2025 illustrates a growing institutional consciousness within public discourse. Furthermore, discussions about “Magnetic Preference” (T09), which is a newly emergent topic, show strong positivity (approximately 77%), reflecting enthusiasm for magnetically aligned and wireless-charging innovations. This finding exemplifies how technology-driven differentiation can rejuvenate market perception even amid saturation.

Collectively, these results suggest that public cognition of portable power banks is evolving toward a multidimensional framework that combines practical, affective, and normative dimensions: the public not only cares about “how well” devices perform but also “how safely,” “how fairly,” and “under which brand reputation” they operate.

This study conducted a comprehensive computational analysis of large-scale social media discourse to elucidate the structure and evolution of public perception and sentiment toward portable power banks in China. By integrating multi-view clustering and LLM-driven opinion evaluation, the research captures both the topic and sentiment states of public engagement with emerging portable power banks.

### 4.2 Comparison with previous studies

The present findings extend and refine earlier research in both conceptual scope and empirical depth.

Compared with the work of Czerniak et al. [8], who identified capacity, price, and charging efficiency as the main predictors of consumer satisfaction, this study demonstrates that public evaluation has broadened beyond physical or technical quality. Users now place equal or greater emphasis on brand credibility, price fairness, and electrical safety, marking a paradigm shift from technical utility to an integrated socio-technical perspective. This shift aligns with the maturation of consumer awareness in energy product markets, where purchase decisions are increasingly mediated by trust, reputation, and perceived risk management rather than mere specification comparison.

In relation to the study by Lu [11], who examined shared power bank adoption among college students, the current analysis both corroborates and extends those findings. Lu observed that although shared rentals were convenient and efficient, cost and risk perception significantly weakened user satisfaction. The present study, using large-scale naturalistic data, confirms that price perception functions as the most decisive emotional trigger in shaping sentiment polarity. Negative emotions concentrate on perceived overpricing and low service transparency, while positive emotions cluster around autonomy and long-term cost savings in self-purchase scenarios. By incorporating both ownership and rental discourse into a unified analytical framework, this study uncovers a semantic bifurcation of energy consumption modes—with self-purchase associated with trust and empowerment, and rental use linked to uncertainty and dependence.

At the industrial level, Sang examined the power-bank sector from a corporate management perspective [9], emphasizing unstable demand, limited profit models, and safety risks as constraints on sustainable development. The current study complements this enterprise-oriented view from the consumer side, revealing that these same structural deficiencies manifest as negative sentiment toward price fairness, value adequacy, and certification reliability. The misalignment between corporate pricing strategies and perceived consumer value emerges as a central mechanism linking micro-level dissatisfaction to macro-level sustainability challenges. Thus, public opinion-derived evidence in this work substantiates and deepens Sang’s diagnosis by providing the affective and perceptual foundation for understanding why specific market reforms, such as price standardization or safety labeling, remain urgently needed.

### 4.3 Theoretical and practical implications

Theoretically, this study advances the scholarly understanding of public cognition and sentiment formation in the context of portable energy technologies by proposing an LLM-driven socio-semantic analytical framework. Through the integration of multi-view clustering and large language model–based opinion mining, the research demonstrates how public discourse on social media embodies a multidimensional evaluative structure that interlinks functional utility, affective judgment, and trust-based perception. The findings reveal that public opinion regarding portable power banks operates within a value–trust–risk triadic mechanism, which extends the theoretical foundations of the Value Adoption Model (VAM) and Technology Acceptance Model (TAM).

Specifically, (1) Perceived usefulness, represented by attributes such as endurance, charging efficiency, and reliability, serves as the cognitive core that shapes the evaluation of the product evaluation; (2) Perceived trust and safety, including brand reputation and certification credibility, function as mediating forces influencing emotional confidence and adoption intention; (3) Perceived fairness, encompassing price rationality and cost–performance balance, regulates satisfaction and long-term loyalty. This triadic configuration conceptualizes user perception as a dynamic equilibrium between instrumental reasoning and affective assessment, bridging the analytical gap between technological evaluation and social cognition. The incorporation of LLM-based semantic modeling further substantiates the interpretive power of computational linguistics in energy-behavior research, demonstrating that social-media expression constitutes a form of collective intelligence that continuously negotiates meaning, trust, and value around emerging portable-energy technologies.

In practice, the study provides actionable information for manufacturers, service providers, and policy makers seeking to enhance consumer confidence, industrial sustainability, and regulatory responsiveness within China’s portable power bank market. (1) Companies should improve production standardization, safety testing, and real-time disclosure of compliance information. Clear and verifiable labeling (e.g., national 3C standards) can reduce perceived safety risks and improve institutional trust; (2) Addressing the persistent misalignment between product cost and user-perceived fairness is crucial. Dynamic, usage-based, or tiered pricing models could improve public sentiment, particularly within the shared-rental sector where dissatisfaction remains high; (3) Continuous technological enhancement, such as magnetic attachment, wireless charging, and lightweight design optimization, can sustain positive emotion and strengthen brand differentiation in a competitive market; (4) Leveraging LLMs and social-media analytics for real-time opinion tracking will allow companies to identify emerging issues promptly, transforming negative sentiment into data-driven product or service improvements; (5) Regulators should coordinate certification standards, rental-service supervision, and transportation-safety regulations to reflect actual public concerns. Establishing unified, transparent governance frameworks will enhance both public trust and market resilience.

It is worth noting that the regulatory environment surrounding the portable power bank industry is tightening rapidly. In November 2025, the Ministry of Industry and Information Technology (MIIT) released the *Safety Technical Specification for Portable Power Banks (Draft for Comments)* and initiated a public consultation process [68], indicating that a new mandatory national standard—stricter than the existing CCC certification framework—will soon be implemented. Industry reports suggest that the draft introduces significantly higher technical requirements in areas such as structural design, circuit-board protection, and battery cell safety. Multiple supply-chain stakeholders anticipate that the compliance difficulty and cost will increase substantially, implying that the market entry threshold for portable power banks is likely to be redefined [69].

In the face of a tightening regulatory environment, our findings reveal a clear convergence between consumer concerns and policy priorities. Safety standards, certification transparency, carrying regulations, and pricing fairness are recurring themes in user discussions and policy texts, revealing a strong structural symmetry between public expectations and regulatory focus. Through the analysis of event-driven spikes in attention and stable, non-event perceptions, the study indicates that regulatory events amplify existing concerns while long-term consumer sentiments provide a substantive basis for policy design. Aligning regulatory actions more closely with these persistent concerns could improve policy responsiveness, reduce uncertainty during transitions, and strengthen overall market trust.

## 5 Conclusion

This study presents a novel LLM-driven analytical framework for examining public opinion on portable power banks in China through large-scale social-media comment mining, identifying 12 discussion topics with satisfactory inter-topic distinctiveness (Topic Redundancy Rate = 0.59) and diversity (Effective Topic Ratio = 1.0). The analysis reveals that public perception of portable power banks is co-constructed through intertwined dimensions of functionality, safety, fairness, and brand trust(10/12 topics, 87.09% of total labels), rather than being determined solely by technical performance, which constitutes only a minor share (12.91%) of overall discussion. By employing multi-view clustering and large language model–assisted sentiment interpretation, the study offers both an empirical understanding of social cognition and a methodological contribution to the integration of artificial intelligence and energy-behavior research.

The results enrich the theoretical discourse on public perception within distributed-energy systems by demonstrating how user-generated content encapsulates multi-dimensional evaluations that merge technological utility with social trust and moral judgment. Empirically, the study provides evidence-based insights for industry practitioners, highlighting that aligning product innovation, transparent pricing, and safety certification with consumer expectations is essential for building sustainable trust. Theoretically, it underscores the transformative potential of AI-assisted semantic modeling in bridging technological development and public perception, thereby offering a replicable framework for future investigations of AI-mediated opinion formation in the domains of portable energy technologies.

Nevertheless, several limitations should be acknowledged. First, the analysis relies primarily on social media data, which may introduce demographic bias toward younger, urban, digitally active users, limiting the generalizability of findings to consumer segments underrepresented on social media, such as older adults, non-users of social platforms, or silent consumers who rarely express opinions online, whose purchasing motivations, usage scenarios, and risk perceptions regarding portable power banks may differ to some extent. Additionally, users who actively post product comments may also constitute a self-selected sample whose expressions tend to concentrate on content with clear-cut attitudes or strong experiential feelings; the sentiment distributions identified in this study may therefore have limited representativeness when generalized beyond this specific user population. Second, the mining paradigm intentionally excluded low-frequency and marginal discussions to enhance signal precision, leaving certain peripheral opinions unrepresented. Third, the analysis did not model platform-level recommendation mechanisms and information diffusion effects. Platform algorithms may tend to promote high-conflict content such as electrical safety incidents or brand trust disputes; since such pushed content inherently carries sentiment bias, the user comments elicited in response are also likely to exhibit corresponding sentiment tendencies, which may have affected the observed sentiment balance and limited the generalizability of findings to non-algorithm-mediated contexts. Additionally, some aspects of sentiment interpretation depend on large language models, which introduces cross-contextual uncertainty in classification outcomes.

Future research could validate this framework’s transferability across diverse data sources and multilingual corpora while incorporating causal modeling and multimodal fusion to capture the deeper mechanisms of public cognitive processes. These enhancements would improve the applicability of this approach in consumer sentiment monitoring, product design optimization, and policy formulation for intelligent, energy-related devices.
